# The wright stuff: reimagining path analysis reveals novel components of the sex determination hierarchy in *drosophila melanogaster*

**DOI:** 10.1186/s12918-015-0200-0

**Published:** 2015-09-04

**Authors:** Justin M. Fear, Michelle N. Arbeitman, Matthew P. Salomon, Justin E. Dalton, John Tower, Sergey V. Nuzhdin, Lauren M. McIntyre

**Affiliations:** Department of Molecular Genetics and Microbiology, University of Florida, CGRC Room 116, PO Box 100266, FL 32610-0266 Gainesville, FL USA; Biomedical Science, Florida State University, Tallahassee, FL USA; Molecular and Computational Biology, University of California, Los Angeles, CA USA

## Abstract

**Background:**

The *Drosophila* sex determination hierarchy is a classic example of a transcriptional regulatory hierarchy, with sex-specific isoforms regulating morphology and behavior. We use a structural equation modeling approach, leveraging natural genetic variation from two studies on *Drosophila* female head tissues – DSPR collection (596 F1-hybrids from crosses between DSPR sub-populations) and CEGS population (75 F1-hybrids from crosses between DGRP/Winters lines to a reference strain w1118) – to expand understanding of the sex hierarchy gene regulatory network (GRN). This approach is completely generalizable to any natural population, including humans.

**Results:**

We expanded the sex hierarchy GRN adding novel links among genes, including a link from *fruitless* (*fru*) to *Sex-lethal* (*Sxl*) identified in both populations. This link is further supported by the presence of *fru* binding sites in the *Sxl* locus. 754 candidate genes were added to the pathway, including the splicing factors *male-specific lethal 2* and *Rm62* as downstream targets of *Sxl* which are well-supported links in males. Independent studies of *doublesex* and *transformer* mutants support many additions, including evidence for a link between the sex hierarchy and metabolism, via *Insulin-like receptor*.

**Conclusions:**

The genes added in the CEGS population were enriched for genes with sex-biased splicing and components of the spliceosome. A common goal of molecular biologists is to expand understanding about regulatory interactions among genes. Using natural alleles we can not only identify novel relationships, but using supervised approaches can order genes into a regulatory hierarchy. Combining these results with independent large effect mutation studies, allows clear candidates for detailed molecular follow-up to emerge.

**Electronic supplementary material:**

The online version of this article (doi:10.1186/s12918-015-0200-0) contains supplementary material, which is available to authorized users.

## Background

**Fig. 1:**
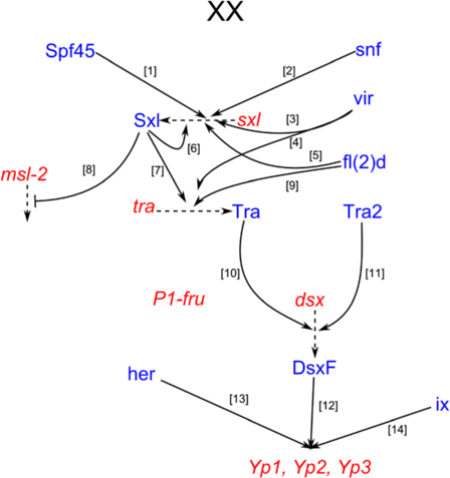
The Drosophila sex determination hierarchy in females. Transcripts are in red and proteins are in blue. Solid arrows are genetic interactions (e.g. splicing, transcription) and dashed arrows are protein translation. (1) Spf45 → Sxl [122], (2) Snf → Sxl [123], (3) vir → Sxl [27], (4) vir → tra [27], (5) fl(2)d → Sxl [10], (6) Sxl → Sxl [122], (7) Sxl → Tra [28, 124], (8) Sxl -| Msl-2 [2, 88, 125], (9) fl(2)d → Tra [126], (10) Tra → DsxF [71], (11) Tra2 → DsxF [71], (12) DsxF → Yps [16, 127, 128], (13) Her → Yps [129], (14) ix → Yps [67]

The *Drosophila melanogaster* sex determination hierarchy consists of an alternative pre-mRNA splicing cascade (Fig. 1) that directs somatic sex differences in morphology reviewed in [1], sex chromosome dosage compensation reviewed in [2], and adult reproductive behaviors reviewed in [3, 4]. Sex differences initiate during early embryogenesis with the sex-specific splicing of *Sex-lethal* (*Sxl*) pre-mRNAs, producing functional Sxl protein in females. Sxl regulates its own pre-mRNA splicing [5] and directs the sex-specific splicing of *transformer* (*tra*) [6]. Female-specific *transformer* (Tra^F^) in conjunction with non-sex-specific *transformer-2* (Tra-2), control all aspects of somatic sex determination by regulating the splicing of the pre-mRNAs that encode the sex-specific transcription factors *doublesex* (*dsx*) [7] and *fruitless* (*fru*) [8], reviewed in [9]. This level understanding of the sex hierarchy has taken several decades of effort [8, 10–16], however the complete gene regulatory network (GRN) is still unknown.

Recent efforts to expand the sex hierarchy GRN have used genomic approaches to characterize global changes in gene expression associated with large-effect mutations in *tra* [17–19], *dsx* [18, 19], and *fru* [19–22]. These studies have identified thousands of novel genes regulated by the sex hierarchy, but how best to incorporate these results into the existing knowledge of the pathway remains elusive.

Here we exploit natural alleles to provide a window into the relationships among genes in the *D. melanogaster* sex hierarchy GRN. Mutation is a driving force of evolution and natural populations are reservoirs of genetic variation. Segregating allelic variation provides many small-effect mutations in regulatory and coding regions of almost every gene reviewed in [23]. Natural variation provides an opportunity to study the impact of genetic variation on the sex hierarchy specifically and more generally on GRNs [24–26]. While the phenotypic consequences of large-effect mutations in the sex hierarchy are well characterized [10, 27–29], the consequences of small-effect mutations are under investigation [24, 30, 31]. For example, genetic variation in upstream splicing factors of the sex hierarchy are known to affect the transcript abundance for downstream targets like *Yolk protein* [26].

Gene expression, from adult female heads, was modeled using two separate transcriptomic datasets from populations of natural alleles. The first was a microarray dataset of 596 F1-hybrids created by crossing recombinant inbred lines (RILs) from each sub-population in the *Drosophila Synthetic Population Resource* (DSPR) [32]. The second was a RNA-seq dataset from (BioProject PRJNA281652) [33]. This CEGS population was made by crossing natural isogenic females from two North American populations of *D. melanogaster* – *Drosophila Genetic Reference Panel* (DGRP; [34]) and Winters California [35] – to males of a laboratory stain (*w*^*1118*^).

A variety of methods have been used to re-construct GRNs including partial correlations [36], differential equations [37], graphical Gaussian networks [38], and Bayesian networks [39–46]. To expand the sex hierarchy GRN, we first built a baseline structural model using the existing molecular knowledge. Then variations of the baseline model were explored and tested using structural equation modeling (SEM) [47]. SEM is a supervised approach based on Sewell Wright’s path analysis [48], where the order and direction of the relationships between genes is an intrinsic part of the structural model.

SEMs not only have been used historically [48, 49], but have also been applied to variety of genetic questions. SEMs have been used to model relationships between QTLs and phenotype in plant development in Arabidopsis [50], grain yield in wheat [51], height and diameter in loblolly pine [52], body size in mouse [53], BMI in humans [54], and others (reviewed by [55]). SEMs have also been used to order *cis-*eQTL [44, 56, 57]. In time course studies, the latent variable structure available in SEMs has been applied to model protein-DNA interactions in yeast [58], transcription in yeast [59], cell lineage determination in *C. elagans* [60], and cell cycle in yeast [61]. They have also been used to construct local GRNs based on patterns of differential gene expression [30, 62]. Here we use the SEM framework to enable GRN expansion. Using existing knowledge about a GRN as a baseline model, we systematically scan the genome for additional components and improve our understanding of the existing GRN in a context that enables interpretation and confirmation. We identified several novel relationships among genes in the sex hierarchy and were able to add novel genes to the sex hierarchy. These new relationships were validated using additional gene expression data from *tra* and *dsx* mutants.

## Results

mRNA isoforms were initially modeled, as the sex hierarchy is regulated by alternative isoforms, but covariation among genes in female head tissue was similar for different isoforms. Therefore, genes in the sex hierarchy were modeled using SEMs and model fit was assessed using four penalized model fit statistics (see Materials and Methods; Additional file 1: Table S1). A baseline model was constructed from the current molecular understanding of the sex hierarchy (Fig. 1). This baseline model was expanded by adding new paths among genes in the sex hierarchy GRN or by adding new genes to all possible locations in the sex hierarchy GRN. Paths that improved model fit compared to the baseline model were considered putative relationships. These models were validated using transcriptomic data from the existing literature and two independent mutation studies for *tra* [17] and *dsx* (presented here).

### Baseline model

**Fig. 2:**
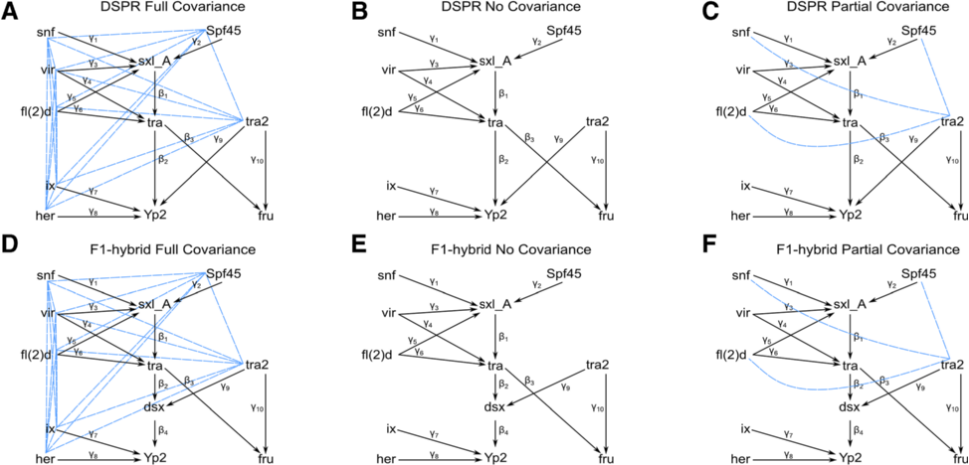
Identification of appropriate covariance structure for baseline model. Three separate covariance models were compared for the DSPR (**a**-**c**) and the CEGS (**d**-**e**). The full covariance model (**a** and **d**) allows all independent, or exogenous, genes to freely co-vary (*blue lines*). The full covariance model implies that there are unknown regulatory factor(s) that is drive expression of the genes in the sex hierarchy GRN. The no covariance model (**b** and **e**) constrains all covariances between exogenous genes to 0. The no covariance implies that the independent genes in the model are truly independent and are not regulated by some unknown factor. The partial covariance model (**c** and **f**) allows exogenous genes to freely covary (*blue lines*). The partial covariance models implies that some of the exogenous genes in the sex hierarchy GRN may be controlled by an unknown factor such as the transcriptional or splicing machinery

The directional path between any two genes in the sex hierarchy was determined from prior molecular knowledge (Fig. 1). For each regulatory relationship identified in the literature, the corresponding directed path parameters β and γ (Fig. 2 black arrows) were estimated in a baseline model. The DSPR and CEGS populations had slightly different baseline GRNs because *dsx* expression was not measured in the DSPR. The *dsx* branch of the sex hierarchy regulates three closely related *Yolk protein* genes (*Yp1*, *Yp2*, and *Yp3*). These three *Yolk protein* genes were highly correlated in both the DSPR and CEGS, to avoid issues of multicollinearity only *Yp2* was included in the baseline model.

Many of the direct relationships between genes in the sex hierarchy have been described, however the covariance patterns among genes are not described. Therefore, three patterns of covariance were compared in the DSPR and CEGS data. (1) The full covariance model estimates covariance parameters between all independent genes (Fig. 2a, d). The full covariance model implies that there is at least one unmeasured factor (e.g., transcription factor or splicing machinery) causing correlation among residual error. The full covariance model simultaneously estimates a matrix of covariance parameters (φ; Fig. 2a, d blue lines) for all pairwise relationships among independent genes. (2) The no covariance model assumes that all independent genes in the pathway are not co-regulated (Fig. 2b, e). The no covariance model constrains all covariance parameters (φ) to 0. (3) In addition to the two extremes, full covariance and no covariance, it is possible to specify a model where some covariance is allowed. A partial covariance model has both co-varying genes and independent genes. Covariance may be due to co-regulation or to hidden effects. The current sex determination literature does not suggest direct co-regulation. However, we saw evidence of co-variation among (*tra2*, *snf*, *Sp45*, and *fl(2)d*) in both DSPR and CEGS data, and use this empirical observation to fit a partial covariance model (Fig. 2c, f). The partial covariance model constrains some covariance parameters of independent genes to 0 while estimating the remaining residual covariance parameters in the SEM (φ’; Fig. 2c, f blue lines).

The three covariance models were compared in both the DSPR and CEGS populations using four penalized fit statistics: adjusted goodness-of-fit (AGFI), parsimonious goodness-of-fit (PGFI), consistent Akaike’s information criterion (CAIC), and Bayesian information criterion (BIC) (Additional file 1: Table S1). All 4 fit statistics selected the no covariance model in the DSPR, while the CEGS results were less clear (Table 1): AGFI selected the full covariance model, PGFI selected the no covariance model, and both CAIC and BIC selected the partial covariance model. An examination of the residual matrix suggest that there are potentially unidentified components of the model. Together with the unambiguous results from the DSPR, this suggest that no covariance model is the most likely, with unknown components present. For these reasons we focus on the no covariance model for the remaining analyses for both DSPR and CEGS.

**Table 1:** Comparison of baseline models using 4 penalizing fit statistics

	DSPR			CEGS		
	Full covariance	No covariance	Partial covariance	Full covariance	No covariance	Partial covariance
Adjusted GFI (AGFI)	0.7953	0.8456^a^	0.8368	0.9895^a^	0.9856	0.9875
Parsimonious GFI (PGFI)	0.3605	0.6993^a^	0.6505	0.468	0.7813^a^	0.7374
CAIC	18196.00	18137.85^a^	18152.94	566.67	562.75	543.93^a^
BIC	18140.00	18102.85^a^	18114.94	507.67	524.75	502.93^a^

^a^indicates the model with the best fit according to the given fit statistics

### Adding new paths to the sex hierarchy GRN

**Fig. 3:**
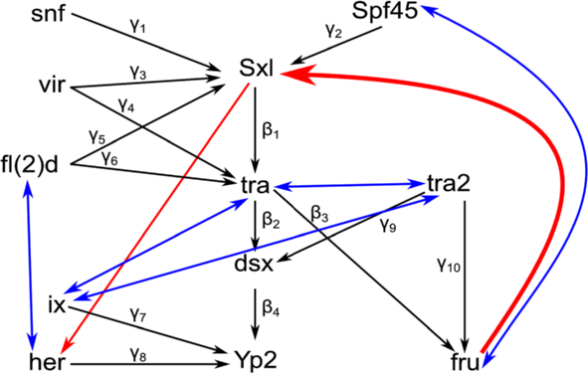
New paths added in both the DSPR and CEGS populations. Directional arrows represent the path between genes. The baseline sex hierarchy SEM is indicated with black arrows and is based on the current understanding of the literature (summarized in Fig. 1). Twelve additional paths improved model fit for both the DSPR and CEGS population compared to their respective baseline models (Fig. 2). Ten of these show bi-directional relationships (*blue arrows*) and two had a single directional relationship (*unidirectional red arrows*)

While much is known about the sex hierarchy, there are potentially unidentified regulatory relationships among the genes in this pathway. Indeed, after accounting for all the known relationships in the sex hierarchy GRN in our baseline model, there were locations in the residual matrix that showed large differences between the estimated and observed values (Additional file 1: Table S3 and S4). Model fit statistics were used to explore these relationships. All possible additional paths – i.e., all possible pairwise interactions between genes in the baseline model excluding interactions already present in the baseline – were individually added to the sex hierarchy and each model fit was compared to the baseline model using BIC. There are 84 possible new paths in the DSPR data and 104 possible new paths in the CEGS data. In the DSPR, 24 paths improved model fit using BIC (Additional file 1: Table S5) and 28 paths improved model fit in the CEGS data (Additional file 1: Table S6). Twelve of these paths overlapped between the DSPR and CEGS (Fig. 3). These 12 paths included five reciprocal relationships (e.g., *fru* → *Spf45* and *fru* ← *Spf45*) and two directed relationships (*fru* → *Sxl* and *Sxl* → *her*). The reciprocal relationships could represent co-regulation, while the directed relationships could represent direct or indirect causal paths (*fru* → *Sxl* or *fru* → gene → *Sxl*). The direct causal path of *fru* → *Sxl* is supported by molecular evidence showing that *Sxl* locus contains *fru* DNA binding sites [20].

### Identifying new genes and adding paths in the sex hierarchy GRN

**Fig. 4:**
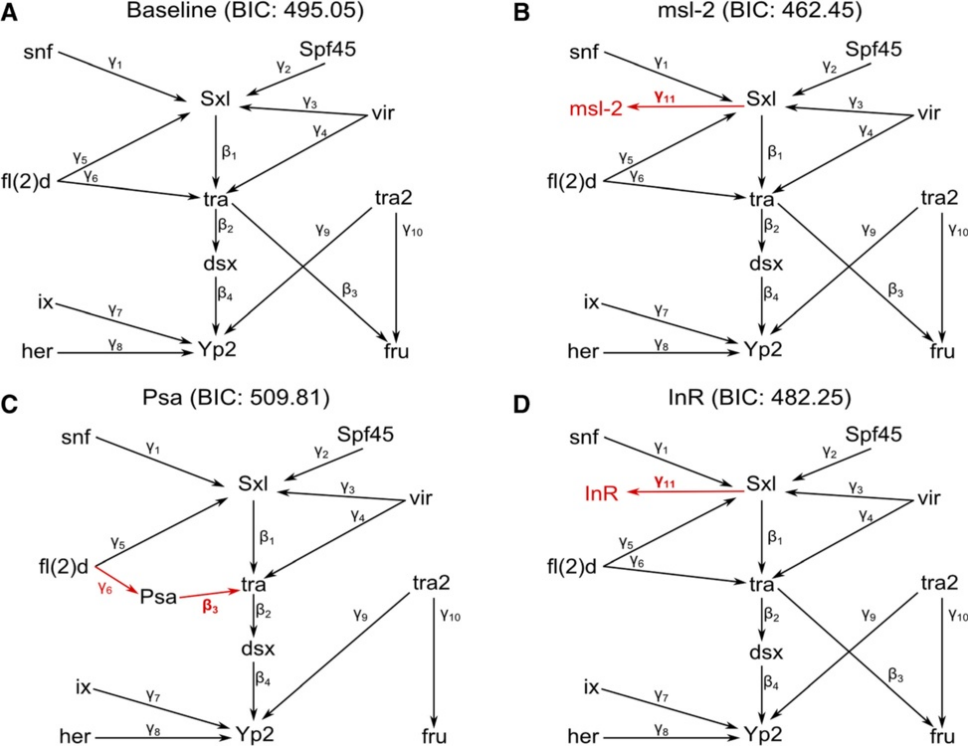
Sex hierarchy expansion. **a** All genes expressed in the CEGS data were added to the baseline model one at a time to all possible paths and model fit was assessed using BIC. For each possible addition the regulatory relationship (*arrows*) has a parameter estimate (γ for exogenous → endogenous and β endogenous → endogenous). There were 754 genes with BIC values lower than the baseline. Some examples include: *msl-2* downstream of *Sxl* **b** *, Psa* in between *tra* and *fru* **c**, and InR downstream of *Sxl* **d**

The sex hierarchy is a splicing and transcriptional regulatory cascade, that affects expression of many genes [17, 19, 20]. To order potential targets in the sex hierarchy, all expressed genes in the DSPR (7,411) and CEGS (8,810) datasets were added one at a time to all possible locations in the sex hierarchy GRN, putative genes were identified by assessing model fit (BIC) compared to the baseline model. The type I error rate of this procedure was estimated via simulation (see Materials and Methods). There were 34 possible locations in the DSPR no covariance baseline model, and 37 possible locations in the CEGS no covariance baseline model. In DSPR, none of the 251,974 tested models improved BIC compared to baseline, while CEGS had 12,565 models (754 genes: Fig. 4, Additional file 1: Table S6) out of 325,970 total tested models (8,810 genes) that improved BIC by more than the level representing a 5 % type I error rate. To determine how sensitive our inferences were to the specification of the covariance model, we repeated the adding genes procedure with the full covariance model for both populations. No genes were added with to the DSPR and 925 genes were added to the CEGS model. The 925 genes added by the full covariance model contained all 754 genes from the no covariance model along with an additional 171 genes (data not shown). We conclude that the results are similar regardless of covariance structure used. In order to determine whether the addition of genes to the baseline model was sensitive to the structure of the model, we used the DSPR baseline model (i.e., without *dsx*) for the CEGS data. Here we found that 98 % of the genes added with the original CEGS baseline model are also added alternate baseline model (i.e., without *dsx*). We also added *msl-2* to the CEGS baseline model and the results from adding genes to this expanded CEGS baseline were also almost identical to the original CEGS baseline model. Finally, we removed *tra* from the original baseline model and identified 95 % of the same genes. It is worth noting that while the vast majority of the same genes are identified, their most likely positions are subject to change. The addition of genes is robust to some deviations in the baseline model specification.

### Validating expanded GRN

The core sex hierarchy GRN terminates with the transcription factors *dsx* and *fru*. New models that incorporated genes downstream of *dsx* (models 3 and 25) or *fru* (model 4) were clear targets for validation studies. There were 329 genes where model 3 (*dsx* → gene) or model 25 (*dsx* → gene → *Yp2*) improved model fit over the baseline model. Of these, 12 genes selected model 3 or model 25 as the most likely model (Table 2). Similarly there were 389 genes where model 4 (*fru* → gene) improved model fit over the baseline model, and five of these genes selected model 4 as the most likely model (Table 3). Reassuringly, four of the five genes identified as most likely to be downstream of *fru* contained a *fru* DNA binding motif [20].

**Table 2:** Genes whose best fitting model was downstream of *dsx*

Symbol	Primary FBgn
lab	FBgn0002522
mei-41	FBgn0004367
CG7099	FBgn0032517
Snap	FBgn0250791
mxc	FBgn0260789
Surf4	FBgn0019925
CG17841	FBgn0028480
CG9328	FBgn0032886
CG7461	FBgn0034432
sec63	FBgn0035771
CG2218	FBgn0039767
Aplip1	FBgn0040281

**Table 3:** Genes whose best fitting model was downstream of *fru*

Symbol	primary_fbgn	Fru binding site^a^
cact	FBgn0000250	1
Aats-asp	FBgn0002069	0
MED15	FBgn0027592	1
CG17162	FBgn0039944	1
Rgk1	FBgn0264753	1

^a^fru binding sites identified in [20]

*dsx* encodes sex-specific transcription factors known to control nearly all aspects of somatic sex differentiation outside of the nervous system, and has a role in the nervous system [63–66]. Sex-specific splicing of *dsx* pre-mRNAs results in a smaller C-terminal region in Dsx^F^ that can interact with the obligate binding partner *intersex* (*ix*) to regulate transcription [67]. Genes affected by *dsx* were identified by comparing differences in gene expression between chromosomally XX *dsx* null animals (*dsx*^*d+R3*^*/dsx*^*m+R15*^) and two strains of wild-type files (Berlin and Canton S). To control for background affects, a gene was considered differentially expressed if an exonic region showed differential expression in the same direction for both control comparisons (Berlin and Canton S), with an FDR ≤ 0.05. There were a total of 340 genes that increased gene expression and 208 genes that decreased gene expression as a result of *dsx* knockout. Of these 208 genes, 13 genes added to the sex hierarchy GRN and were enriched for DSX binding sites ( = 0.0015) (Table 4).

**Table 4:** Genes added to sex hierarchy GRN that were also repressed by *dsx* knockout

FBgn	Symbol	BIC^a^	*fru* binding site^b^	*dsx* binding site^c^
FBgn0051635	CG31635	482.4517	1	0
FBgn0259111	Ndae1	484.761	1	0
FBgn0038659	endoA	489.4473	1	0
FBgn0031885	Mnn1	491.0274	1	0
FBgn0063649	CG6006	495.2368	1	0
FBgn0053144	CG33144	497.0297	1	0
FBgn0031390	tho2	499.8877	0	0
FBgn0030608	Lsd-2	503.0053	1	0
FBgn0037890	CG17734	503.0885	0	0
FBgn0011224	heph	503.2395	1	1
FBgn0037252	CG14650	509.1222	1	0
FBgn0037466	CG1965	509.3656	1	0
FBgn0013984	InR	511.9545	1	1

^a^Improve fit indicated by smaller BIC than baseline (524.7484) - 12

^b^Genes containing *fru* binding sites from [20]

^c^Genes containing *dsx* binding sites [95]

### Comparison with unsupervised approaches

**Fig. 5:**
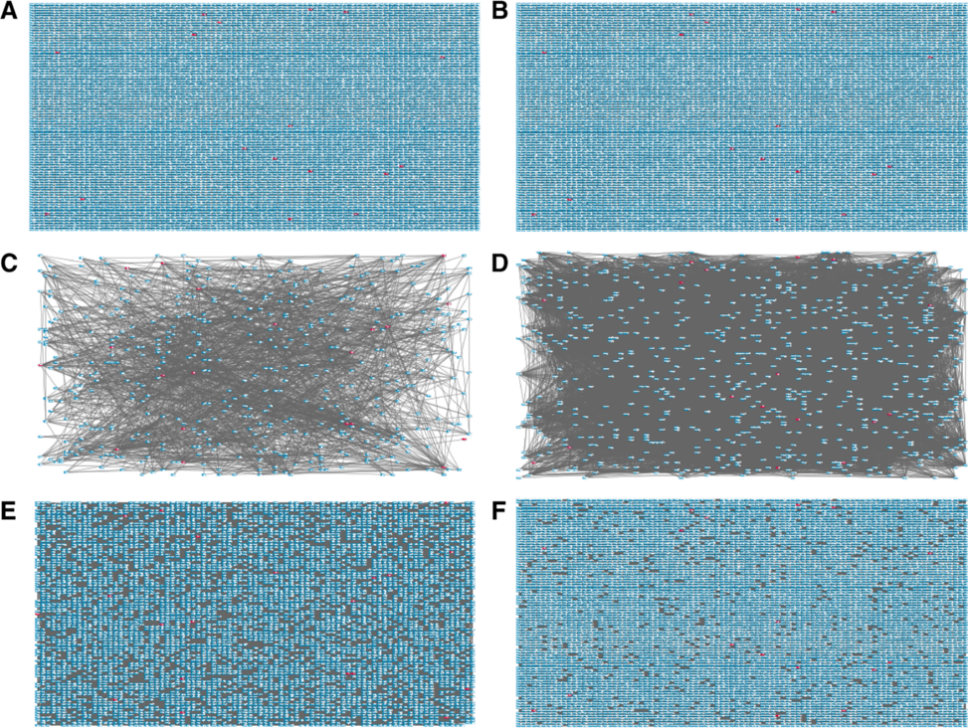
Genome-wide graphical Gaussian network of genes in the DSPR and CEGS. Red boxes represent genes that are part of the sex hierarchy or associated splicing factors. Blue boxes are remaining genes in the DSPR (**a**, **c**, **e**) or CEGS (**b**, **d**, **f**). (**a**-**b**) Visualization of the genome-wide GGN. (**c**-**d**) The primary neighborhood 1-step out from sex determination genes. (**e**-**f**) The secondary neighborhood 2-steps out from sex determination genes

Similar to supervised approaches, unsupervised GRNs can be re-constructed from genome-wide expression studies. Unsupervised GRNs reflect the correlation structure in the gene expression data, often suggesting novel connections and thus facilitate hypothesis generation. There are many methods for the re-construction of unsupervised genome-wide GRNs [68–70]. Here we use the *GeneNet* package [70] to build an unsupervised GRN using graphical Gaussian networks (GGN). The GGN of the DSPR (Fig. 5a) and CEGS data (Fig. 5b) show no obvious clustering among genes in the sex hierarchy, independent of whether the network was constructed using data from genes or transcript isoform (data not shown). To improve visualization, nearest neighborhoods were created by focusing on sub-networks of genes 1-step and 2-steps away from genes in the sex hierarchy. Again, no obvious clustering of genes in the sex hierarchy is evident in either the DSPR (Fig. 5c, e) or CEGS data (Fig. 5d, f). Finally, the sex hierarchy regulatory structure could not be captured by only focusing genes in the sex hierarchy (DSPR: Additional file 1: Figure S1).

Isoforms of the same gene were reasonably close in the estimated network. For example, when looking at the nearest neighborhood sub-network for any of the *fru* isoforms, all 15 *fru* isoforms were within two steps in both the DSPR and the CEGS datasets (fru_PD; Additional file 1: Figure S2). The sex hierarchy specific GGN also clusters isoforms from the same gene in the DSPR data (Additional file 1: Figure S1). Transcript isoforms are expected to share expression regulatory mechanisms including common transcription factors, therefore highly correlated expression patterns are not surprising between isoforms.

The lack of proximity among genes known to be part of the core sex hierarchy was disappointing. Most genes were not within two steps of each other with few exceptions. (Spf45_PA; Additional file 1: Figure S2). GGNs show some plausible novel relationships. For example, the DSPR GGN shows a putative novel relationship *tra* and *tra2* (tra_PA; Additional file 1: Figure S2). In females, TRA and TRA2 form a protein complex that regulates *dsx* and *fru* splicing [71]. Protein-protein interactions are not necessarily expected to be re-capitulated in GRNs, but transcriptional co-regulation of *tra* and *tra2* makes biological sense. GGNs also identify a large number of genes (grey nodes) not previously associated with the sex hierarchy, though they show strong expression correlations to members of the sex hierarchy. These genes are candidates to further examine. This unsupervised network re-construction did not robustly detect known relationships among genes in the sex hierarchy.

## Discussion

*Drosophila* lines from natural populations contain a reservoir of genetic variation, with each line containing a unique combination of numerous mutations of small to moderate effect. For instance, among seven *D. simulans* natural genotypes, 80 % of proteins have at least one allele with altered protein sequence [72]. These alleles are viable in the wild, implying that they are unlikely to be large-effect deleterious mutations. Furthermore, mutations in regulatory regions are likely to result in differences in gene expression between any two genotypes. For example, when two different *Drosophila* lines are compared, approximately 10 % of genes exhibit transcript abundance changes of 1.3-fold or larger [73–76]. Gene expression is also highly heritable [77, 78] and there is abundant genetic variation in the transcript level of all sex determination genes [30, 79]. Therefore, a collection of a several dozen unrelated lines is expected to contain regulatory mutations in almost every gene.

Genetic variation in the context of a GRN contributes to phenotypic differences in complex traits [41, 80–85] and natural variation is a powerful tool to expand knowledge of GRNs [24, 25, 82]. The number of regulatory relationships that can be elucidated by any model, is expected to depend upon the number of observable allelic combinations in the population. The sex hierarchy is an excellent target for proof of principle studies, with relatively few genes in the core pathway (*n* = 12) and there is extensive knowledge about the relationships among these genes. This prediction was borne out by our analysis, where the allelic combinations in the DSPR and CEGS dataset readily identified possible new paths among genes already in the sex hierarchy (Table 5).

**Table 5:** The number of alleles per gene of the sex determination hierarchy in the DSPR and CEGS populations

chr	Start	Stop	Gene	FBname	CGnum	DSPR	CEGS (assume ref)	CEGS (imputed)
2 L	16677185	16679691	her	FBgn0001185	CG4694	11	28	16
2 L	22959606	22961118	Spf45	FBgn0086683	CG17540	4	11	7
2R	7246942	7247668	ix	FBgn0001276	CG13201	4	9	7
2R	9707129	9712058	fl(2)d	FBgn0000662	CG6315	13	23	13
2R	10489509	10491842	tra2	FBgn0003742	CG10128	NA	14	12
2R	12748826	12755219	Psi	FBgn0014870	CG8912	NA	37	7
2R	19247715	19253878	vir	FBgn0003977	CG3496	NA	62	44
3 L	16583159	16584150	tra	FBgn0003741	CG16724	4	8	6
3 L	21837888	21925802	mub	FBgn0262737	CG7437	NA	16	6
3R	3750045	3793130	dsx	FBgn0000504	CG11094	NA	21	3
3R	5243662	5281222	ps	FBgn0261552	CG42670	NA	29	10
3R	6610105	6612187	Rbp1	FBgn0260944	CG17136	4	5	NA
3R	9460584	9472026	sqd	FBgn0263396	CG16901	NA	10	6
3R	9487033	9492613	B52	FBgn0004587	CG10851	12	17	6
3R	14239995	14371308	fru	FBgn0004652	CG14307	15	73	40
X	5203275	5204534	snf	FBgn0003449	CG4528	8	16	13
X	6968583	6992089	Sxl	FBgn0264270	CG43770	NA	7	5
X	9944984	9946669	Yp2	FBgn0005391	CG2979	9	23	12
X	9947844	9949531	Yp1	FBgn0004045	CG2985	9	20	9
X	13653579	13655580	Yp3	FBgn0004047	CG11129	14	37	17

NA: Indicates that depth of coverage was insufficient to estimate the number of alleles

In the CEGS population, the core sex determination pathway was expanded. While in the DSPR there was not enough evidence to add genes to the core pathway. These two populations differ in their origin and construction and there are several possible explanations for this discrepancy. The inclusion of *dsx* in the CEGS data where it was not detected in the DSPR, is the most obvious possibility. This is unlikely to be the whole explanation as there were only 12 genes that selected downstream of *dsx* as their most likely model. We also used the DSPR baseline model (without *dsx*) and found that 98 % of the genes added in the original CEGS baseline model we added to the DSPR baseline model using the CEGS data. Taken together, this evidence suggests that the absence of *dsx* in the DSPR is not driving the lack of addition of new genes to the DSPR data. Another possibility is the amount of allelic variation at each locus. The CEGS data have almost twice the number of alleles at each locus, while the sample size is smaller. To test this idea a random 50 % subset of CEGS lines were used and the modeling process repeated with this subset. The number of alleles, and the results were nearly identical in this subset (Additional file 1: Table S7) further suggesting that the number of alleles is more important than the number of lines. Another possibility is the presence of *trans*-effects. There is evidence for *trans*- effects among these genes in *D. simulans* [86]. Since the DSPR have a much higher degree of diversity among the alleles, the *trans*- effects in the DSPR may be larger than in CEGS, increasing the noise relative to the signal [87].

One of the struggles in genomic studies is how to validate associations. The DSPR and the CEGS population are two independent populations, created from different starting populations and assayed using different technologies. The intersection of 12 additional links lends strength to these associations, as it is unlikely that the same spurious results would be found in two independent populations. In particular, the *fru* to *Sxl* link has additional support with the existence of *fru* binding sites in the regulatory region of *Sxl* [20]. The question of how to validate the addition of genes is more complex, as the DSPR with fewer alleles did not show evidence for GRN expansion. However, the sex hierarchy has been the subject of much exploration; therefore, it is possible to use the accumulation of knowledge from previous studies for validation of novel links suggested by the SEM models as well as the results from knock-out, knock down and overexpression studies.

Genes identified by GRN expansion show previous evidence of sex-biased differential expression, regulation by the sex determination hierarchy, and sex-biased splicing. There were 178 genes in the expanded GRN that have previously been shown to have sex-biased differential expression upstream or downstream of *tra* [17, 19]. One of these sex-biased expressed genes, *msl-2*, is known to be regulated by *Sxl* in males [2, 88]. The inclusion of *msl-2* improved model fit over baseline in all models, and the most likely model just upstream or just downstream of *Sxl* (Fig. 4b). The expanded GRN was also enriched for genes with evidence of sex-biased splicing [89] (*p* < 0.0001). For example, *Rm62* improved model fit over baseline in all models, and has been shown to have sex-biased splicing in whole bodies [89]. Given that the sex hierarchy is a sex-specific splicing cascade it was not surprising to find genes associated with B52 splicing [90] were enriched in the expanded GRN (*p* = 0.0117).

Of the 754 genes identified as co-regulated with the sex determination GRN, there was enrichment for chromatin binding (*p* = 0.05) and helicase activity (*p* = 0.006) after FDR correction (Additional file 1: Table S8). Genes associated with these GO terms are consistent with a role in sex determination. The chromatin genes include transcriptional regulators of the homeotic and polycomb group (PcG) that are involved in producing sex-specific adult morphology, such as *mxc* and *ph-p*; as well as regulators of dosage compensation. Dosage compensation is the component of sexual differentiation that yields a similar ratio of X-chromosome to autosome gene expression in males and females [91, 92]. In *Drosophila* males, the proteins MSL1, MSL2, and MSL3 combine with the helicase MLE to form a chromatin-binding complex called MSL. In females, SXL protein inhibits formation of MSL complex by inhibiting the translation of the *msl-2* mRNA. In our female experiment, *msl-2* is linked to *Sxl*. MSL titrates the histone acetylase MOF away from the autosomes, thereby reducing autosomal gene expression. MSL also modulates MOF activity on the X to produce a net two-fold increase in X chromosome gene expression relative to the autosomes. Regulation of gene expression by MSL and MOF involves the PcG group genes and chromatin modifying complexes, including helicases. The identification of chromatin binding genes and helicase genes is logical in this context. Interestingly, the genes that are identified on the chromatin list also include components of the piRNA pathway, which regulates transposon expression and chromatin structure in the germ-line cells. The piRNA pathway was recently shown to regulate sex-determination in the silkworm *Bombyx mori* [93] and these genes are logical candidates for expansion of the sex hierarchy GRN. Finally, components of the nonsense-mediated decay (NMD) pathway, which is involved in regulation of gene expression at the level of RNA, including the sex-determination gene Tra [94] were also identified.

In addition to the use of the literature as validation, we conducted a mutational study for validation purposes where *dsx* null mutants were examined. Genes directly regulated by *dsx* should contain a *dsx* binding site. Of the 13 genes added by GRN expansion and differentially expressed in the *dsx* null experiment, *InR* and *heph* contained a *dsx* binding site [95] suggesting that these may be direct targets of *dsx*.

The present GRN analysis suggests a connection between *InR* and *Sxl,* (Fig. 4d). However, this relationship may be more complicated given that there are also *dsx* binding sites in the *InR* locus. The lack of consistent differences in *InR* transcript abundance between males and females in head tissue supports the link found by the SEM between *Sxl* and *InR* rather than a direct link from *dsx* to *InR*. In *Drosophila*, *Sxl* is the master switch of the sex hierarchy. *Sxl* is an RRM-type RNA binding protein [96], that is involved in splicing and translational regulation reviewed in [9]. According to current gene models, *InR* isoforms do not show exon skipping, indicating that the putative *Sxl* action is binding to the 3’UTR and influencing mRNA stability or translation efficiency. There is previous indirect evidence for a connection between the sex hierarchy and metabolism; *Drosophila* females are larger than males and *InR* is required for the sex-dimorphism in size [97]. Furthermore, studies have also found an association between natural variation in *InR* and body size [31]. Changes in insulin signaling also affects re-mating behavior [98]. The *Drosophila* fat body, analogous to adipose tissues, is important for courtship behaviors [19, 99] and the *fru*-expressing neurons that underlie courtship behavior are obesity blocking neurons [100].

Natural allelic variation provides an elegant way to estimate and potentially order relationships in a GRN. Large scale unsupervised approaches quickly run out of degrees of freedom and are often difficult to interpret in the context of the existing literature. In the experiments presented here, unsupervised methods identified isoforms as belonging to the same gene, but failed to cluster genes in the core sex hierarchy and did not yield many associations that were able to be verified in the existing literature. In contrast, a supervised SEM approach combined with a genome-wide scan of expressed genes leads to logical expansions of the GRN. Novel relationships were validated by their presence in two independent experiments, the DSPR and the CEGS. Genes that are part of the putative GRN expansion suggest that the sex determination pathway is involved with sex-biased splicing in adult female heads and further than the pathway’s expansion is significantly enriched for components likely to help mediate this sex-biased splicing. These results here also support recent literature suggestion that transcription and splicing are part of one continuous process reviewed in [101, 102] and that the sex hierarchy in adult females is an active part of this process.

Unlike with unsupervised approaches, GRNs constructed based on existing understanding of a particular provide testable hypotheses. The promise of this approach is that it can be applied directly to human populations where population level expression studies can be modeled with SEM and cell culture experiments can be used to test new links.

## Conclusions

Understanding the biological processes by which genotype contributes to phenotype, has as a logical step along the way of understanding how genes interact with each other in a regulatory framework. Here we use a novel method which combines classical molecular genetic approaches (i.e., large-effect mutation studies) with populations of natural alleles to not only identify but order transcriptional regulatory interactions. Like other population genetic studies, the number of alleles limits the number of observable interactions and differences in population panel design will have different strengths and weaknesses. Here we not only identify novel transcriptional relationships among genes within the sex hierarchy GRN (i.e., *fru* → *Sxl*), but identified 754 candidate genes to add to the existing pathway structure. These genes were enriched for sex-biased splicing, components of the spliceosome, chromatin factors, and helicase activity. Many of the genes added to the sex hierarchy GRN have previous evidence in the literature for a connection, which the SEM models confirm and/or were supported by the *dsx* mutation study. Intriguingly, a connection between the sex hierarchy and metabolism is suggested here with the identification of *InR* as associated with this pathway.

## Methods

### Naturally derived populations

The DSPR F1-hybrids were constructed by crossing individuals from each sub-population in the DSPR [32]. Briefly, the DSPR population captures global genetic variation by creating two RIL sub-populations using 15 highly inbred founder strains derived from wild-caught *D. melanogaster* from around the world. These 15 founder strains were randomly split into two subpopulations, each with eight strains (one line was shared between populations). Subpopulations were maintained separately for 50 generations, when mating pairs were selected and total of ~1700 RILs were created [103]. To create the DSPR F1-hybrids, King et al. crossed females from population **pA** to males from population **pB** for a total of 596 F1-hybrids.

The CEGS F1-hybrid population was created by crossing 75 natural isogenic strains to a single laboratory strain (w^1118^) as described by (BioProject PRJNA281652) [33]. The 75 natural isogenic strains were derived from two North American populations: the *Drosophila Genetic Resource Population* (DGRP) from Raleigh North Carolina [34], and a second population from Winters California [35].

### Allelic variation

We compared the amount of allelic variation present in the starting genotypes used in the DSPR and CEGS populations. The number of alleles present in the 15 founder genotypes of the DSPR was determined by extracting all SNP calls (DSPR founder’s data Release 3; [104]) that intersected the CDS regions of genes in the sex hierarchy. Similarly, we extracted all SNP calls that intersected the CDS regions of the sex hierarchy genes from 75 genotypes used in the CEGS population. For both data sets, SNP calls at every variable position within the CDS regions of a given gene were assigned to an individual and for each individual the number of unique SNP combinations across CDSs regions was counted as the number of alleles for a gene. The number of alleles per gene for both data sets is summarized in (Table 5). Two different filtering criteria were used for the CEGS SNP calls, 1) All filtered SNP calls with missing genotypes assumed to be reference and 2) All filtered SNP calls with an additional MAF filter of 5 % and imputed missing genotypes.

### Gene expression

GRN models were constructed using two previously published datasets: the DSPR: [32] and CEGS. For the DSPR study, pre-processed gene expression data from female head tissue were downloaded from [32]. Briefly, King et al., isolated RNA from 596 F1-hybrids from a pool of 250–300 adult female heads. Global gene expression was measured using Nimbelgen 12 x 135 k arrays (16,637 transcripts with eight 60 bp probes per transcript). For the CEGS study, pre-processed gene expression data from female head tissues were obtained from [33]. Briefly, Kurmangaliyev et al., isolated RNA from 75 F1-hybrids from a pool of 50 adult female heads. Global gene expression was measured using HiSeq 2000 on at least 3 biological replicates for each F1-hybrid.

### Examination of isoforms

The sex hierarchy is a splicing cascade. Many of the genes in the sex hierarchy are alternatively spliced to give rise to sex specific transcript and protein isoforms. A common splicing strategy seen in many genes of the sex hierarchy is the inclusion of premature stop codons resulting in non-functional proteins. There are also sex specific alteration of 3’ and 5’ UTRs in some of the genes in the hierarchy, sex specifically changing mRNA processing efficiency. Given the importance of isoform usage in the sex hierarchy several methods were used to determine if covariation among genes was driven by variation in isoform expression.

Patterns of variation in the correlation between isoforms, exons, or genes within the sex hierarchy GRN were identified with factor analysis. The FACTOR procedure (SAS v9.3) was used with the PRINCIPAL method and VARIMAX orthogonal rotation. For each dataset, the number of factors was selected using scree plots and the proportion of explained variation. Isoform level factor analysis of the DSPR identified a total of 16 factors. For each gene, the majority of isoforms loaded on the same factor, indicating that isoforms do not drive variation at the level of the gene (Additional file 1: Table S9). Isoforms were summarized to the gene level and a second factor analysis selected 8 factors (Additional file 1: Table S10). While each factor did not seem to represent a specific part of the sex hierarchy, many of the splicing factors loaded together, and the *yolk proteins* also loaded together (Additional file 1: Table S10). The CEGS population showed similar results with a total of 17 factors identified when analyzing exons, again the majority of exons within a gene loaded together (Additional file 1: Table S11). Variation in exonic correlation did not inform variation in correlation between genes. Exons were collapsed to the gene level and factor analysis identified 12 factors with good gene separation (Additional file 1: Table S12).

Modulated modularity clustering (MMC) was also used to group related genes into modules [105]. Similar to factor analysis MMC uses variance-covariance structure to identify relationships. MMC identified 23 modules in the DSPR isoform data (Additional file 1: Table S13) and 2 modules in the exon level CEGS population (Additional file 1: Table S14). Variation in gene expression was not driven by variation between isoforms or exons, therefore gene level expression was used. The DSPR and CEGS captured expression for most genes in the sex hierarchy, but the DSPR experiment did not capture *dsx* or *msl-2* expression.

### Structural equation models (SEM)

Path analysis, a predecessor to SEM, was first introduced by the geneticist Sewall Wright [48, 49]. Path analysis relates observed covariances to parameters in a structural model, these parameters represent direct and indirect causal interactions between of variables and can then be estimated from the data. In the GRN framework, the structure of a transcriptional regulatory pathway (i.e., splicing/transcriptional relationships between genes) is modeled (Fig. 1). Here, each gene is a node in the network and causal relationships between genes are directional paths. The directional paths between genes can be thought of as regression coefficients. Within this model there are two classes of genes: independent genes (*fl(2)d*, *her*, *ix*, *snf*, *Spf45*, *tra2*, and *vir*) have no paths leading into them (i.e., can be thought of as x’s in regression), and dependent genes (*dsx*, *fru*, *Sxl*, *tra*, and *Yp2*) have at least one path going into them (i.e., can be thought of as y’s in regression). Following the general notation of SEM [47] we write the SEM for the sex hierarchy as:

1 $$ \eta =B\eta +\varGamma \xi +\zeta $$

where *B*_5 × 5_ is the coefficient matrix for the relationship between dependent variables, *Γ*_5 × 7_ is the coefficient matrix for the relationship between independent and dependent variables, *η*_5 × 1_ is the vector of dependent variables, *ξ*_7 × 1_ is the vector of independent variables, and *ζ*_5 × 1_ is the vector of random errors. In addition, there are two covariance matrices: *Φ* the covariance matrix for ξ and *Ψ* the covariance matrix for ζ. Maximum Likelihood estimation is then used to solve for the parameters by minimizing the difference between the covariance matrix implied by the structural model and the covariance matrix of the data (Bollen). SEMs require a large number of samples and assume the data follow a multivariate normal distribution [106]. The DSPR data set contains a large number of samples (*n* = 596) and expression levels of data in the sex hierarchy are approximately normally distributed (Additional file 1: Table S3A). The CEGS population has a moderate number of samples (*n* = 75) and while RNA-seq data are based on counts, after log transformation and upper quartile normalization the genes in the sex hierarchy in the CEGS data are also approximately normal (Additional file 1: Table S3B)**.** RNA-seq data have been modeled by others as normal after transformation (e.g. [107–109]). Results were consistent with a number of different normalization strategies, including standardization. We also looked at the covariance/correlation structure of genes in the sex hierarchy and found that DSPR has weaker correlations among genes in the sex hierarchy while having a more variation (Additional file 1: Figure S4).

#### Identifiability

A key consideration for any network re-construction method is the identifiability of the model. A model is identified if it is possible to derive a unique estimate for every model parameter. Identifiability is a property of the model and not the data, therefore it can be (and should be) ascertained prior to data collection [106]. If a model is not identified, it is impossible to solve for unique estimates, regardless of the amount of data collected. A structural model is identified if all of the unknown parameters can be formulated as functions of the known parameters and that these functions can lead to unique solutions [47]. This is an algebraic exercise where unknown parameter (i.e., B’s, Γ’s, Φ’s, Ψ’s) are solved in terms of the variance and covariance matrix Σ. We refer the reader to [47, 106, 110] for a detailed discussion on what makes a model identifiable. The sex determination hierarchy is an ideal case for identification, it is a linear cascade, with few known feedback loops (only *Sxl* autoregulation). To maintain identifiability in our baseline model (below), we assume that residual errors of the endogenous variables do not co-vary.

#### Goodness of fit

Various model fit statistics have been developed to measure how well observed data fit the covariance structure implied by the structural model [47, 106, 110]. This has the advantage of simultaneously evaluating all of the relationships in the GRN and is analogous to evaluating the overall model fit of a linear model. Three well established model fit criteria – AIC [111], CAIC [112], and BIC [113] are considered. The goal of AIC is to select the best approximating model or set of models supported by the data. CAIC is a small sample version of AIC which should be used when sample size is much smaller than the number of parameters being estimated [114]. In contrast, BIC will asymptotically select the ‘true’ model, assuming the ‘true’ model is in the set. All of these criteria (AIC, CAIC, and BIC) allow comparison and ranking of models to separate those that are equally useful from those that are not [114]. These criteria are also flexible enough to be used as an overall fit index or as an incremental fit index (Additional file 1: Table S1).

### Development of a baseline model

We fit three separate baseline models using the CALIS procedure (SAS v9.3) for both the DSPR and CEGS data. The baseline model is based on the imperfect understanding we currently have of the sex hierarchy. It is not only possible, but likely that other similar models would fit the data at least equally as well as the model derived from the current literature. In addition, we are uncertain about the appropriate covariance structure. We examined this initial baseline model with different covariance structures. The full covariance model (Fig. 2a, d) defined the structural model based on the sex hierarchy pathway, and estimated parameters for each path and all covariances between independent genes. The no covariance model (Fig. 2b, e) was the same as the full covariance model, except that covariances between independent genes were set to 0. The partial covariance model (Fig. 2c, f) was the same as the no covariance model except the covariances between: *tra2* ↔ *snf*, *tra2* ↔ *Spf45*, and *tra2* ↔ *fl(2)d* were estimated.

As with any model selection and model specification process, there are likely to be issues of model equivalence. That is, there are likely to be several alternative formulations of the model that are equivalent in their ability to describe the data. Indeed, as with any modeling approach, it is important to keep in mind that the models employed are likely incorrect and to assess several alternate formulations to determine the impact of model specification has on the downstream inferences.

### GRN expansion

The GRN was expanded by adding paths one-at-a-time either as new links among genes within the sex hierarchy or as new genes in various locations within the sex hierarchy. A custom python module (SEMNET) was created to write the CALIS (SAS v9.3) code for each of these models. First, this module takes a representation of the pathway of interest and generates CALIS code adding all possible new links among genes within the pathway. Second a list of expressed genes not in the pathway, and CALIS code will be generated adding these genes to all possible locations in the pathway. SAS was then used to run all models and BIC scores were analyzed to determine which models improve fit compared to a baseline model.

### Type I error rate

Data were simulated from both the DSPR and CEGS populations. Genes within the baseline sex hierarchy GRN were simulated by first fitting the baseline SEM with the R package *lavaan* [115] and then using these parameter estimates with the R package *simsem* [116] to generate simulated data with the correct mean and covariance structure. A gene was simulated from a normal distribution with a mean and variances drawn at random with replacement from the population (DSPR or CEGS). This process was repeated 8,000 times. Random genes whose BIC was lower than the simulated baseline represent type I errors. For the CEGS data 95 % of the genes with BIC values lower than the baseline had a difference of less than 12. The simulation was repeated and the BIC threshold was almost identical. We then used a difference in BIC of 12 as a 5 % type I error cutoff to determine whether to add a gene to the model with the real data for the CEGS population. For the DSPR, no simulated genes were found to have BIC values lower than the baseline in the DSPR data, suggesting the model might be saturated. To allow for the possibility of real data being more informative than the random data, we used a cutoff of a BIC difference of 0 for the DSPR data. The type I error simulation can also indicate when a model is potentially underfit. Using the non-transcriptionally regulated pathway (InR/tor), the corresponding type I error simulations had the opposite result, with almost all random genes showing BIC values lower than the baseline, suggesting that the baseline model for this pathway has little information in it and can be improved by the addition of almost any gene. These type I error simulations not only allow us to control for the type I error in adding genes to the network, but also provide an window into the baseline model fit, and can indicate when baseline models are potentially underfit (InR/tor) or overfit (DSPR).

### DSPR and CEGS GRN expansion

Using GRN expansion, all possible new paths were added among genes within the sex hierarchy GRN for both the DSPR and CEGS. A total of 84 (104) new models were created for the DSPR (CEGS) data. Each model was evaluated and model fit was compared to the no covariance baseline model. All expressed genes not in the sex hierarchy GRN were then added to all possible paths. A total of 34 (37) locations in the GRN that a gene could be added to DSPR (CEGS) data. Each model was evaluated and model fit was compared to the no covariance baseline model. A difference of 12 BIC was required to consider the model different from baseline.

### *dsx null mutant* for *GRN validation*

A large-scale perturbation in *dsx* was created as a molecular validation. Transcriptome libraries were prepared from adult heads in three independent biological replicates for each of the following strains: Canton S females, Berlin females, and *dsx*^*d+R3*^*/dsx*^*m+R15*^ females. All flies were collected 0 to 16 h post-eclosion under CO_2_ anesthetization and allowed to recover for 8 h before being snap frozen in liquid nitrogen. Snap frozen whole animals used for head collections were stored at −80 °C until head were collected.

Adult heads were separated from bodies by mechanical tapping of the cryovial. A piece of plastic was cooled on dry ice, on which the heads were separated from the bodies and immediately transferred to TRIzol®. Approximately 200 heads were harvested per biological replicate of each genotype, and homogenized in 1 mL of Trizol. Total RNA was extracted using TRIzol® protocol (Invitrogen, Carlsbad, CA), with the following modification to the precipitation step: precipitate using 250 μL Isopropanol and 250 μL 1.2 M NaCitrate, 0.8 M NaCl in DEPC-treated H2O. Total RNA was DNase (Ambion) treated to remove any trace amounts of DNA. Poly(A) + transcripts were isolated subsequently using MicroPoly(A) Purist™ Kit (Ambion). To facilitate quality control of reads across our samples, at this stage of library construction we spiked-in small amounts of exogenous RNA from ArrayControl™ Kit (Ambion) into 100 ng poly(A) + RNA. Spike-in control sequences selected had similar lengths (~1 kb), had no significant alignment to the *D. melanogaster* transcriptome using 25 base pair alignments, and had no significant alignment among the other spike-in sequences chosen. Five spike-in controls (Ambion ArrayControl™ RNA Spike 3 through 7) were added to each of our 12 samples in decreasing amounts following a log2 scale. The combination of spike-in controls represented, on average, 0.08 % of the total RNA pool for each sample. 100 ng of Poly(A) + transcripts were fragmented for 3 min 50 s at 70 °C to approximately 250 base pairs by chemical fragmentation (Ambion). First strand cDNA was synthesized using SuperScript® II Reverse Transcriptase (Invitrogen) and a combination of random hexamer and oligo (dT) primers. Second strand cDNA was synthesized using DNA polymerase I in combination with ribonuclease H (NEB). Double stranded cDNA templates were blunt ended using End-It™ Repair Kit (Epicentre), and A-overhangs were added at both ends with Klenow fragment (3' → 5' exo-minus). Illumina sequencing adapters were then ligated to both ends of the cDNA templates using Fast-Link™ DNA Ligation Kit (Epicentre). We then enriched for cDNA templates by performing multiplex incorporating PCR reactions (≤18 cycles), and isolating 250–550 base pair fragments by gel purification. During PCR, unique index sequences (Illumina) were incorporated into each biological sample to allow identification of reads from each sample when multiple samples were sequenced on a single lane of the flow cell. All samples were run on a 72 base pair single end flow cell. Images were processed using Illumina's GenomeStudio software. On average ~6 million reads were obtained per sample.

Reads were mapped and analyzed as in [20]. A total of 44,585 exonic regions were detected at least once in each treatment group and were assessed for differential gene expression. A linear model was fit for each exonic region separately, and all comparisons were done using contrasts with a single model with a FDR correction [117]. To reduce the chances of a strain effect, all treatment groups were compared to 2 control strains (Berlin and Canton S). Exonic regions were summarized to the gene level. A gene was considered differentially expressed if it had an exonic region with a FDR ≤ 0.2 in both control comparisons. Fold-change direction was used to designate a gene as repressed or induced.

### GRN validation

New paths identified using GRN re-construction need to be independently validated. Instead of performing an individual experiment for each new path, genomic data can be harnessed for validation. First, DNA binding site studies can be used to support direct molecular interactions between the upstream and downstream genes. Three previous studies identified putative DNA binding sites for *fru* [20], *dsx* [95], and *tra* [17]. Second, single-gene perturbation studies can be used to identify changes in patterns of global gene expression, identifying gene directly or indirectly regulated by the perturbed gene. Two previous studies looked at the effects of perturbing *tra* in female heads [17, 19], and a new study presented here looks at the effects of *dsx* perturbation in female heads. New paths identified by GRN expansion were validated by looking for support for the relationship in DNA binding sites and single-gene perturbation studies.

### Unsupervised approaches

Graphical Gaussian networks (GGN) are a popular method to infer gene network structure [118–121]. An unsupervised approach, GGNs use partial-correlation to infer conditional dependency, allowing the construction of a hypothetical network without prior knowledge of its structure. We constructed two GGNs, one genome-wide and one based on the sex hierarchy GRN, were constructed using the R package *GeneNet* [38] for both the DSPR and the CEGS populations. Edges were selected using either an FDR cutoff of 0.2 or a number cutoff of 20 edges.

### Availability of supporting data

RNA-seq data for the *dsx* null experiment is available at the Gene Expression Omnibus (GSE67400). All other datasets have been previously published, please see citations for more information.

### Software availability

SAS PROC CALIS was used for all SEM analysis. A python package (SEMNET) was created to generate adding links and adding genes PROC CALIS statements (http://github.com/McIntyre-Lab/semnet). All scripts related to the analysis of this project can be found at http://github.com/McIntyre-Lab/papers/tree/master/fear_sem_sd_2015.

## Additional file

Additional file 1:
**Table S1.** Structural equation modeling measures of overall model fit. Adapted from (Hoyle). **Table S2.** DSPR no covariance raw residual matrix of genes in the sex hierarchy GRN. **Table S3.** CEGS no covariance raw residual matrix of genes in the sex hierarchy GRN. **Table S4.** New links added between genes in the DSPR sex hierarchy GRN. **Table S5.** New links added between genes in the CEGS sex hierarchy GRN. **Table S6.** Gene added to the CEGS sex hierarchy GRN. **Table S7.** Number of alleles per gene of the sex determination hierarchy in a random 50 % subset of CEGS lines. **Table S8.** Genes added to the sex hierarchy GRN that showed enrichment for chromatin binding and helicase activity. **Table S9.** DSPR isoform level factor analysis. **Table S10.** DSPR gene level factor analysis. **Table S11. ** CEGS exonic regions factor analysis. **Table S12.** CEGS gene level factor analysis. **Table S13.** DSPR isoform level modulated modularity clustering (MMC). **Table S14.** CEGS exon level modulated modularity clustering (MMC). **Figure S1.** Graphical Gaussian network of genes in the sex determination hierarchy (DSPR Collapsed Isoforms). **Figure S2.** Examples of secondary neighborhood structure from a genome-wide graphical Gaussian network. **Figure S3.** Distribution of genes in the sex hierarchy. **Figure S4.** Correlation and covariance matrices for genes in the sex hierarchy. (DOCX 1153 kb).

## Abbreviations

AGFI: Adjusted goodness-of-fit
AIC: Akaike’s information criterion
BIC: Bayesian information criterion
CAIC: Consistent Akaike’s information criterion
DGRP: Drosophila Genetic Reference Panel
DSPR: Drosophila Synthetic Population Resource
Dsx: Doublesex
eQTL: Expression quantitative trait loci
FDR: False discovery rate
fl(2)d: Female lethal d
fru: Fruitless
GGN: Graphical Gaussian Networks
GO: Gene ontology
GRN: Gene regulatory network
Heph: Hephaestus
Her: Hermaphrodite
InR: Insulin-like Receptor
Ix: Intersex
MMC: Modulated modularity clustering
MOF: Males absent on the first
msl-2: Male-specific lethal 2
mxc: Multi sex combs
NMD: Nonsense-mediated decay
PcG: Polycomb group
PGFI: Parsimonious goodness-of-fit
ph-p: Polyhomeotic proximal
QTL: Quantitative trait loci
RIL: Recombinant inbred line
SEM: Structural equation modeling
Snf: Sans fille
SNP: Single nucleotide polymorphisms
Spf45: Splicing factor 45
Sxl: Sex-lethal
Tor: Target of rapamycin
Tra: Transformer
tra2: Transformer 2
vir: Virilizer
Yp: Yolk protein
