# Supplementary material for: The wright stuff: reimagining path analysis reveals novel components of the sex determination hierarchy in drosophila melanogaster
Source: BMC Syst Biol. 2015 Sep 4;9:53. doi: 10.1186/s12918-015-0200-0 (PMC4558766; doi:10.1186/s12918-015-0200-0)
Supplement: Additional file 1: — Table S1. Structural equation modeling measures of overall model fit. Adapted from (Hoyle). Table S2. DSPR no covariance raw residual matrix of genes in the sex hierarchy GRN. Table S3. CEGS no covariance raw residual matrix of genes in the sex hierarchy GRN. Table S4. New links added between genes in the DSPR sex hierarchy GRN. Table S5. New links added between genes in the CEGS sex hierarchy GRN. Table S6. Gene added to the CEGS sex hierarchy GRN. Table S7. Number of alleles per gene of the sex determination hierarchy in a random 50 % subset of CEGS lines. Table S8. Genes added to the sex hierarchy GRN that showed enrichment for chromatin binding and helicase activity. Table S9. DSPR isoform level factor analysis. Table S10. DSPR gene level factor analysis. Table S11. CEGS exonic regions factor analysis. Table S12. CEGS gene level factor analysis. Table S13. DSPR isoform level modulated modularity clustering (MMC). Table S14. CEGS exon level modulated modularity clustering (MMC). Figure S1. Graphical Gaussian network of genes in the sex determination hierarchy (DSPR Collapsed Isoforms). Figure S2. Examples of secondary neighborhood structure from a genome-wide graphical Gaussian network. Figure S3. Distribution of genes in the sex hierarchy. Figure S4. Correlation and covariance matrices for genes in the sex hierarchy. (DOCX 1153 kb). [file 12918_2015_200_MOESM1_ESM.docx]

# Tables

**Supplementary Table S1. Structural equation modeling measures of overall model fit. Adapted from (Hoyle 2012).**

| **Fit index** | **Reference** | **Range** | **Sensitive to N** | **Penalty**  **Complexity** | **Direction that is better** |
| --- | --- | --- | --- | --- | --- |
| $GFI=1- \frac{e^{'}We}{s^{'}Ws}$ | Joreskog & Sorbom (1981) | 0-1 | Yes | No | Bigger |
| $AGFI=1- \frac{p^{*}}{df}\left( 1-GFI \right)$ | Joreskog & Sorbom (1981) | 0-1 | Yes | Yes | Bigger |
| $GFI^{*}= \frac{p}{p+2\left( \frac{\chi^{2}-df}{N-1} \right)}$ | Maiti & Mukherjee 1990); Steiger (1989) | 0-1 | No | No | Bigger |
| $AGFI^{*}=1-\frac{p^{*}}{df}\left( 1-GFI^{*} \right)$ | Maiti & Mukherjee 1990); Steiger (1989) | 0-1 | No | Yes | Bigger |
| $CFI=1- \frac{max(\left( N-k \right)f_{min}-{df}_{min},0)}{max(\left( N-k \right)f_{0}-{df}_{0},0)}$ | Bentler (1995) | 0-1 | No | Yes | Bigger |
| $AIC=f+2k$ | Akaike (1974) |  | Yes | Yes | Smaller |
| $CAIC=f+\left( \ln\left( N \right)+1 \right)k$ | Bozdogan (1987) |  | Yes | Yes | Smaller |
| $BIC=f+\ln\left( N \right)k$ | Schwarz (1978) |  | Yes | Yes | Smaller |

**Supplementary Table S2. DSPR no covariance raw residual matrix of genes in the sex hierarchy GRN.**

|  | Spf45 | Sxl | Yp2 | fl_2_d | fru | her | ix | snf | tra | tra2 | vir |
| --- | --- | --- | --- | --- | --- | --- | --- | --- | --- | --- | --- |
| Spf45 | 0 | -0.00245 | 0.10157 | 0.08472 | -0.24911 | 0.01741 | 0.20848 | 0.00382 | -0.01893 | 0.01115 | -0.00036 |
| Sxl | -0.00245 | 0.00016 | -0.04142 | -0.00272 | -0.06643 | 0.08514 | 0.02439 | -0.00259 | 0.00023 | 0.08966 | -0.0011 |
| Yp2 | 0.10157 | -0.04142 | -0.00056 | 0.06731 | 0.04037 | 0.00714 | 0.01014 | 0.16055 | -0.00654 | -0.0004 | 0.02184 |
| fl_2_d | 0.08472 | -0.00272 | 0.06731 | 0 | -0.05838 | -0.11247 | -0.04215 | 0.07961 | -0.00378 | 0.03851 | 0.04346 |
| fru | -0.24911 | -0.06643 | 0.04037 | -0.05838 | 0.00324 | -0.17033 | -0.18645 | 0.04154 | -0.00932 | 0.02145 | 0.01452 |
| her | 0.01741 | 0.08514 | 0.00714 | -0.11247 | -0.17033 | 0 | 0.06289 | -0.01392 | 0.20241 | -0.04776 | -0.11177 |
| ix | 0.20848 | 0.02439 | 0.01014 | -0.04215 | -0.18645 | 0.06289 | 0 | -0.02287 | 0.25537 | -0.18176 | 0.04791 |
| snf | 0.00382 | -0.00259 | 0.16055 | 0.07961 | 0.04154 | -0.01392 | -0.02287 | 0 | 0.0758 | 0.09515 | 0.02065 |
| tra | -0.01893 | 0.00023 | -0.00654 | -0.00378 | -0.00932 | 0.20241 | 0.25537 | 0.0758 | 0.00037 | -0.12315 | -0.0021 |
| tra2 | 0.01115 | 0.08966 | -0.0004 | 0.03851 | 0.02145 | -0.04776 | -0.18176 | 0.09515 | -0.12315 | 0 | -0.0374 |
| vir | -0.00036 | -0.0011 | 0.02184 | 0.04346 | 0.01452 | -0.11177 | 0.04791 | 0.02065 | -0.0021 | -0.0374 | 0 |

**Supplementary Table S3. CEGS no covariance raw residual matrix of genes in the sex hierarchy GRN.**

|  | Spf45 | Sxl | Yp2 | dsx | fl_2_d | fru | her | ix | snf | tra | tra2 | vir |
| --- | --- | --- | --- | --- | --- | --- | --- | --- | --- | --- | --- | --- |
| Spf45 | 0 | -0.0193 | 0.02233 | 0.01271 | -0.01061 | -0.03972 | 0.00929 | 0.03747 | 0.01759 | 0.00941 | 0.00421 | -0.00391 |
| Sxl | -0.0193 | 0.0146 | -0.06119 | -0.01558 | 0.01606 | 0.10121 | 0.03271 | -0.05564 | -0.00813 | -0.00352 | 0.01385 | -0.0039 |
| Yp2 | 0.02233 | -0.06119 | 0.00161 | 0.00086 | -0.0392 | -0.01489 | -0.00816 | 0.00457 | 0.03218 | 0.00856 | 0.0199 | 0.0234 |
| dsx | 0.01271 | -0.01558 | 0.00086 | -0.00079 | -0.00651 | -0.01416 | -0.01139 | 0.00326 | -0.0006 | -0.00303 | 0.00367 | 0.00081 |
| fl_2_d | -0.01061 | 0.01606 | -0.0392 | -0.00651 | 0 | 0.04821 | 0.02635 | -0.01919 | -0.02428 | -0.001 | -0.01317 | 0.0034 |
| fru | -0.03972 | 0.10121 | -0.01489 | -0.01416 | 0.04821 | 0.00069 | 0.01493 | -0.03538 | -0.04153 | -0.00242 | -0.00328 | 0.01433 |
| her | 0.00929 | 0.03271 | -0.00816 | -0.01139 | 0.02635 | 0.01493 | 0 | -0.01329 | -0.00112 | -0.00402 | 0.01743 | 0.00875 |
| ix | 0.03747 | -0.05564 | 0.00457 | 0.00326 | -0.01919 | -0.03538 | -0.01329 | 0 | 0.0457 | 0.05206 | 0.03621 | -0.02249 |
| snf | 0.01759 | -0.00813 | 0.03218 | -0.0006 | -0.02428 | -0.04153 | -0.00112 | 0.0457 | 0 | 0.03088 | -0.00627 | 0.01381 |
| tra | 0.00941 | -0.00352 | 0.00856 | -0.00303 | -0.001 | -0.00242 | -0.00402 | 0.05206 | 0.03088 | 0.00043 | 0.02506 | 0.00003 |
| tra2 | 0.00421 | 0.01385 | 0.0199 | 0.00367 | -0.01317 | -0.00328 | 0.01743 | 0.03621 | -0.00627 | 0.02506 | -0.00724 | 0.00841 |
| vir | -0.00391 | -0.0039 | 0.0234 | 0.00081 | 0.0034 | 0.01433 | 0.00875 | -0.02249 | 0.01381 | 0.00003 | 0.00841 | 0 |

**Supplementary Table S4. New links added between genes in the DSPR sex hierarchy GRN.**

| **Model ID** | **Model Number** | **New Path** | **BIC** |
| --- | --- | --- | --- |
| Model_Baseline | 0 | Baseline | 18102.85 |
| Model_5 | 5 | fru→Sxl | 18100.74 |
| Model_8 | 8 | Spf45→fru | 18059.55 |
| Model_13 | 13 | her→fru | 18097.29 |
| Model_14 | 14 | her→tra | 18087.74 |
| Model_16 | 16 | ix→fru | 18097.19 |
| Model_17 | 17 | ix→tra | 18068.16 |
| Model_18 | 18 | snf→Yp2 | 18092.6 |
| Model_21 | 21 | tra2→Sxl | 18097.99 |
| Model_22 | 22 | tra2→tra | 18097.49 |
| Model_26 | 26 | fru→Spf45 | 18063.3 |
| Model_30 | 30 | Sxl→her | 18100.78 |
| Model_31 | 31 | fru→her | 18090.7 |
| Model_32 | 32 | tra→her | 18084.49 |
| Model_34 | 34 | fru→ix | 18086.87 |
| Model_35 | 35 | tra→ix | 18069.19 |
| Model_36 | 36 | Yp2→snf | 18093.56 |
| Model_39 | 39 | Sxl→tra2 | 18098.34 |
| Model_40 | 40 | tra→tra2 | 18098.75 |
| Model_45 | 45 | ix→Spf45 | 18079.64 |
| Model_50 | 50 | her→fl_2_d | 18100.35 |
| Model_56 | 56 | fl_2_d→her | 18100.35 |
| Model_61 | 61 | Spf45→ix | 18079.64 |
| Model_65 | 65 | tra2→ix | 18086.33 |
| Model_76 | 76 | ix→tra2 | 18086.33 |

**Supplementary Table S5. New links added between genes in the CEGS sex hierarchy GRN.**

| **Model ID** | **Model Number** | **New Path** | **BIC** |
| --- | --- | --- | --- |
| Model_Baseline | 0 | Baseline | 524.7484 |
| Model_3 | 3 | Sxl→fru | 483.7359 |
| Model_9 | 9 | fru→Sxl | 517.207 |
| Model_15 | 15 | Spf45→fru | 519.4891 |
| Model_19 | 19 | fl_2_d→fru | 497.634 |
| Model_27 | 27 | ix→tra | 506.0027 |
| Model_30 | 30 | snf→fru | 516.1672 |
| Model_31 | 31 | snf→tra | 519.6844 |
| Model_34 | 34 | tra2→tra | 520.6226 |
| Model_40 | 40 | fru→Spf45 | 518.4382 |
| Model_44 | 44 | fru→fl_2_d | 501.2978 |
| Model_45 | 45 | Sxl→her | 522.3158 |
| Model_49 | 49 | Sxl→ix | 521.6655 |
| Model_52 | 52 | tra→ix | 507.8206 |
| Model_55 | 55 | fru→snf | 515.2271 |
| Model_56 | 56 | tra→snf | 517.5631 |
| Model_59 | 59 | tra→tra2 | 521.5706 |
| Model_70 | 70 | her→fl_2_d | 513.0123 |
| Model_72 | 72 | snf→fl_2_d | 522.7258 |
| Model_76 | 76 | fl_2_d→her | 513.0123 |
| Model_84 | 84 | snf→ix | 519.542 |
| Model_85 | 85 | tra2→ix | 520.7516 |
| Model_86 | 86 | vir→ix | 521.0022 |
| Model_88 | 88 | fl_2_d→snf | 522.7258 |
| Model_90 | 90 | ix→snf | 519.542 |
| Model_91 | 91 | tra2→snf | 508.3838 |
| Model_96 | 96 | ix→tra2 | 520.7516 |
| Model_97 | 97 | snf→tra2 | 508.3838 |
| Model_102 | 102 | ix→vir | 521.0022 |

**Supplementary Table S6. Gene added to the CEGS sex hierarchy GRN.**

| **primary_fbgn** | **symbol** |
| --- | --- |
| FBgn0000038 | nAcRbeta-64B |
| FBgn0000054 | Adf1 |
| FBgn0000057 | adp |
| FBgn0000097 | aop |
| FBgn0000146 | aub |
| FBgn0000212 | brm |
| FBgn0000244 | by |
| FBgn0000250 | cact |
| FBgn0000257 | car |
| FBgn0000307 | chif |
| FBgn0000317 | ck |
| FBgn0000370 | crc |
| FBgn0000411 | D |
| FBgn0000442 | Pkg21D |
| FBgn0000449 | dib |
| FBgn0000497 | ds |
| FBgn0000499 | dsh |
| FBgn0000542 | ec |
| FBgn0000557 | Ef1alpha100E |
| FBgn0000567 | Eip74EF |
| FBgn0000579 | Eno |
| FBgn0000629 | E(z) |
| FBgn0000635 | Fas2 |
| FBgn0000636 | Fas3 |
| FBgn0000711 | flw |
| FBgn0001075 | ft |
| FBgn0001084 | fy |
| FBgn0001087 | g |
| FBgn0001169 | H |
| FBgn0001206 | Hmr |
| FBgn0001215 | Hrb98DE |
| FBgn0001259 | in |
| FBgn0001325 | Kr |
| FBgn0001330 | kz |
| FBgn0001341 | l(1)1Bi |
| FBgn0001624 | dlg1 |
| FBgn0001941 | ifc |
| FBgn0001961 | Arpc1 |
| FBgn0001978 | stc |
| FBgn0001995 | mRpL4 |
| FBgn0002069 | Aats-asp |
| FBgn0002522 | lab |
| FBgn0002552 | lin |
| FBgn0002673 | twe |
| FBgn0002709 | mei-218 |
| FBgn0002715 | mei-S332 |
| FBgn0002781 | mod(mdg4) |
| FBgn0002878 | mus101 |
| FBgn0002887 | mus201 |
| FBgn0002891 | mus205 |
| FBgn0002906 | Blm |
| FBgn0002909 | mus312 |
| FBgn0002914 | Myb |
| FBgn0002940 | ninaE |
| FBgn0003134 | Pp1alpha-96A |
| FBgn0003178 | PyK |
| FBgn0003204 | ras |
| FBgn0003205 | Ras85D |
| FBgn0003231 | ref(2)P |
| FBgn0003261 | Rm62 |
| FBgn0003334 | Scm |
| FBgn0003371 | sgg |
| FBgn0003416 | sl |
| FBgn0003475 | spir |
| FBgn0003486 | spo |
| FBgn0003612 | Su(var)2-10 |
| FBgn0003687 | Tbp |
| FBgn0003715 | CG16778 |
| FBgn0003716 | tkv |
| FBgn0003882 | tub |
| FBgn0003885 | alphaTub84D |
| FBgn0003890 | betaTub97EF |
| FBgn0003892 | ptc |
| FBgn0003963 | ush |
| FBgn0004049 | yrt |
| FBgn0004101 | bs |
| FBgn0004103 | Pp1-87B |
| FBgn0004168 | 5-HT1A |
| FBgn0004177 | mts |
| FBgn0004227 | nonA |
| FBgn0004363 | porin |
| FBgn0004367 | mei-41 |
| FBgn0004369 | Ptp99A |
| FBgn0004374 | neb |
| FBgn0004380 | Klp64D |
| FBgn0004381 | Klp68D |
| FBgn0004391 | shtd |
| FBgn0004406 | tam |
| FBgn0004432 | Cyp1 |
| FBgn0004509 | Fur1 |
| FBgn0004514 | Oct-TyrR |
| FBgn0004574 | Rop |
| FBgn0004583 | ex |
| FBgn0004643 | mit(1)15 |
| FBgn0004698 | mus210 |
| FBgn0004861 | ph-p |
| FBgn0004864 | hop |
| FBgn0004868 | Gdi |
| FBgn0004876 | cdi |
| FBgn0004896 | fd59A |
| FBgn0004901 | Prat |
| FBgn0004907 | 14-3-3zeta |
| FBgn0004913 | Gnf1 |
| FBgn0004914 | Hnf4 |
| FBgn0004957 | por |
| FBgn0005558 | ey |
| FBgn0005616 | msl-2 |
| FBgn0005624 | Psc |
| FBgn0005630 | lola |
| FBgn0005638 | slbo |
| FBgn0005640 | Eip63E |
| FBgn0005649 | Rox8 |
| FBgn0005671 | Vha55 |
| FBgn0005777 | PpD3 |
| FBgn0008651 | lbl |
| FBgn0010105 | comm |
| FBgn0010194 | Wnt5 |
| FBgn0010238 | Lac |
| FBgn0010303 | hep |
| FBgn0010348 | Arf79F |
| FBgn0010380 | AP-1-2beta |
| FBgn0010391 | Gtp-bp |
| FBgn0010406 | RNaseX25 |
| FBgn0010416 | TH1 |
| FBgn0010488 | NAT1 |
| FBgn0010548 | Aldh-III |
| FBgn0010750 | atms |
| FBgn0011020 | Sas-4 |
| FBgn0011202 | dia |
| FBgn0011211 | blw |
| FBgn0011224 | heph |
| FBgn0011336 | OstStt3 |
| FBgn0011592 | fra |
| FBgn0011655 | Med |
| FBgn0011676 | Nos |
| FBgn0011708 | Syx5 |
| FBgn0011740 | alpha-Man-II |
| FBgn0011741 | Arp6 |
| FBgn0011754 | PhKgamma |
| FBgn0011802 | Gem3 |
| FBgn0011836 | Taf2 |
| FBgn0013263 | Trl |
| FBgn0013276 | Hsp70Ab |
| FBgn0013718 | nuf |
| FBgn0013726 | pnut |
| FBgn0013764 | Chi |
| FBgn0013984 | InR |
| FBgn0013987 | MAPk-Ak2 |
| FBgn0014002 | Pdi |
| FBgn0014006 | Pk92B |
| FBgn0014010 | Rab5 |
| FBgn0014028 | SdhB |
| FBgn0014073 | Tie |
| FBgn0014143 | croc |
| FBgn0014184 | Oda |
| FBgn0014340 | mof |
| FBgn0014366 | noi |
| FBgn0015024 | CkIalpha |
| FBgn0015075 | Ddx1 |
| FBgn0015129 | DopR2 |
| FBgn0015278 | Pi3K68D |
| FBgn0015279 | Pi3K92E |
| FBgn0015295 | shark |
| FBgn0015391 | glu |
| FBgn0015400 | kek2 |
| FBgn0015402 | ksr |
| FBgn0015509 | lin19 |
| FBgn0015542 | sima |
| FBgn0015615 | Cap |
| FBgn0015721 | king-tubby |
| FBgn0015776 | nrv1 |
| FBgn0015790 | Rab11 |
| FBgn0015797 | Rab6 |
| FBgn0015803 | RtGEF |
| FBgn0015949 | hrg |
| FBgn0016080 | xmas-1 |
| FBgn0016641 | PTP-ER |
| FBgn0016696 | Pitslre |
| FBgn0016978 | snRNP-U1-70K |
| FBgn0017418 | ari-1 |
| FBgn0017550 | Rga |
| FBgn0017572 | Mo25 |
| FBgn0019925 | Surf4 |
| FBgn0019947 | Psn |
| FBgn0019960 | CG6455 |
| FBgn0019990 | Gcn2 |
| FBgn0020224 | Cbl |
| FBgn0020238 | 14-3-3epsilon |
| FBgn0020304 | drongo |
| FBgn0020307 | dve |
| FBgn0020391 | Nrk |
| FBgn0020412 | JIL-1 |
| FBgn0020497 | emb |
| FBgn0020503 | CLIP-190 |
| FBgn0020506 | Amyrel |
| FBgn0020510 | Abi |
| FBgn0020620 | RN-tre |
| FBgn0020626 | Osbp |
| FBgn0020647 | KrT95D |
| FBgn0020653 | Trxr-1 |
| FBgn0020910 | RpL3 |
| FBgn0021818 | cnk |
| FBgn0021979 | l(2)k09913 |
| FBgn0022023 | eIF-3p40 |
| FBgn0022097 | Vha36-1 |
| FBgn0022153 | l(2)k05819 |
| FBgn0022268 | KdelR |
| FBgn0022702 | Cht2 |
| FBgn0022768 | Pp2C1 |
| FBgn0022942 | Cbp80 |
| FBgn0022985 | qkr58E-2 |
| FBgn0023001 | melt |
| FBgn0023094 | cyc |
| FBgn0023143 | Uba1 |
| FBgn0023177 | Pp4-19C |
| FBgn0023216 | Parg |
| FBgn0023509 | mip130 |
| FBgn0023511 | Edem1 |
| FBgn0023513 | CG14803 |
| FBgn0023514 | CG14805 |
| FBgn0023536 | CG3156 |
| FBgn0024150 | Ac78C |
| FBgn0024182 | waw |
| FBgn0024238 | Fim |
| FBgn0024291 | Sir2 |
| FBgn0024329 | Mekk1 |
| FBgn0024362 | CG11412 |
| FBgn0024366 | CG11409 |
| FBgn0024432 | Dlc90F |
| FBgn0024807 | DIP1 |
| FBgn0024833 | AP-1mu |
| FBgn0024947 | NTPase |
| FBgn0024989 | CG3777 |
| FBgn0025186 | ari-2 |
| FBgn0025390 | Mur2B |
| FBgn0025608 | Faf |
| FBgn0025628 | CG4199 |
| FBgn0025633 | CG13366 |
| FBgn0025637 | skpA |
| FBgn0025638 | Roc1a |
| FBgn0025674 | CycK |
| FBgn0025743 | mbt |
| FBgn0025781 | Cdc16 |
| FBgn0025790 | TBPH |
| FBgn0025802 | Sbf |
| FBgn0025830 | IntS8 |
| FBgn0026179 | siz |
| FBgn0026181 | rok |
| FBgn0026323 | Tak1 |
| FBgn0026326 | Mad1 |
| FBgn0026369 | Sara |
| FBgn0026427 | Su(var)2-HP2 |
| FBgn0026432 | Grip163 |
| FBgn0026573 | CG8290 |
| FBgn0026634 | ldlCp |
| FBgn0026737 | CG6171 |
| FBgn0026761 | Trap1 |
| FBgn0026777 | Rad23 |
| FBgn0027090 | Aats-gln |
| FBgn0027338 | Kap-alpha3 |
| FBgn0027375 | RecQ5 |
| FBgn0027490 | D12 |
| FBgn0027493 | CG17273 |
| FBgn0027498 | CG2061 |
| FBgn0027503 | CG11970 |
| FBgn0027504 | CG8878 |
| FBgn0027509 | CG7261 |
| FBgn0027514 | CG1024 |
| FBgn0027518 | CG7609 |
| FBgn0027528 | CG9634 |
| FBgn0027532 | CG7139 |
| FBgn0027547 | CG1927 |
| FBgn0027548 | nito |
| FBgn0027558 | pgant3 |
| FBgn0027567 | CG8108 |
| FBgn0027568 | Cand1 |
| FBgn0027569 | cert |
| FBgn0027587 | CG7028 |
| FBgn0027589 | CG1688 |
| FBgn0027592 | MED15 |
| FBgn0027596 | CG10249 |
| FBgn0027607 | CG8230 |
| FBgn0027608 | CG2082 |
| FBgn0027620 | Acf1 |
| FBgn0027844 | CAH1 |
| FBgn0028325 | l(1)G0334 |
| FBgn0028336 | l(1)G0255 |
| FBgn0028341 | l(1)G0232 |
| FBgn0028343 | l(1)G0222 |
| FBgn0028387 | chm |
| FBgn0028401 | Snap24 |
| FBgn0028427 | Ilk |
| FBgn0028433 | Ggamma30A |
| FBgn0028471 | Nab2 |
| FBgn0028474 | CG4119 |
| FBgn0028480 | CG17841 |
| FBgn0028527 | CG18507 |
| FBgn0028538 | Sec71 |
| FBgn0028539 | CG31731 |
| FBgn0028647 | CG11902 |
| FBgn0028665 | VhaAC39-1 |
| FBgn0028671 | Vha100-1 |
| FBgn0028689 | Rpn6 |
| FBgn0028691 | Rpn9 |
| FBgn0028692 | Rpn2 |
| FBgn0028695 | Rpn1 |
| FBgn0028962 | Aats-ala-m |
| FBgn0028979 | tio |
| FBgn0029114 | Tollo |
| FBgn0029672 | CG2875 |
| FBgn0029686 | CG2941 |
| FBgn0029711 | Usf |
| FBgn0029801 | CG15771 |
| FBgn0029822 | CG12236 |
| FBgn0029824 | CG3726 |
| FBgn0029870 | Marf |
| FBgn0029891 | Pink1 |
| FBgn0029893 | CG14442 |
| FBgn0029975 | CG1444 |
| FBgn0029992 | Upf2 |
| FBgn0030003 | CG2116 |
| FBgn0030082 | HP1b |
| FBgn0030089 | AP-1gamma |
| FBgn0030114 | CG17754 |
| FBgn0030141 | Gga |
| FBgn0030206 | CG2889 |
| FBgn0030245 | CG1637 |
| FBgn0030293 | CG1737 |
| FBgn0030330 | Tango10 |
| FBgn0030354 | Upf1 |
| FBgn0030366 | Usp7 |
| FBgn0030451 | CG15717 |
| FBgn0030505 | NFAT |
| FBgn0030506 | Lig4 |
| FBgn0030514 | CG9941 |
| FBgn0030556 | mRNA-cap |
| FBgn0030608 | Lsd-2 |
| FBgn0030625 | CG5877 |
| FBgn0030699 | CG8578 |
| FBgn0030701 | CG16952 |
| FBgn0030734 | CG9911 |
| FBgn0030740 | CG9917 |
| FBgn0030761 | CG9784 |
| FBgn0030833 | CG8915 |
| FBgn0030838 | CG5445 |
| FBgn0030854 | CG8289 |
| FBgn0030858 | IntS2 |
| FBgn0030872 | Ucp4A |
| FBgn0030890 | CG7536 |
| FBgn0031047 | Rcd-1 |
| FBgn0031077 | CG15618 |
| FBgn0031100 | CG1504 |
| FBgn0031150 | bves |
| FBgn0031159 | CG11566 |
| FBgn0031161 | CG15445 |
| FBgn0031187 | CG14619 |
| FBgn0031194 | CG17598 |
| FBgn0031285 | CG3662 |
| FBgn0031319 | CG4896 |
| FBgn0031374 | CG7337 |
| FBgn0031390 | tho2 |
| FBgn0031456 | Trn-SR |
| FBgn0031483 | CG9641 |
| FBgn0031544 | CG17593 |
| FBgn0031571 | CG3921 |
| FBgn0031590 | CG3702 |
| FBgn0031659 | CG14043 |
| FBgn0031713 | CG7277 |
| FBgn0031799 | Pez |
| FBgn0031834 | CG13766 |
| FBgn0031842 | Tango1 |
| FBgn0031879 | uif |
| FBgn0031883 | CG11266 |
| FBgn0031885 | Mnn1 |
| FBgn0031976 | CG7367 |
| FBgn0031990 | CG8552 |
| FBgn0032138 | CG4364 |
| FBgn0032156 | CG13124 |
| FBgn0032157 | Etl1 |
| FBgn0032169 | CG4709 |
| FBgn0032170 | CG4658 |
| FBgn0032243 | Klp31E |
| FBgn0032258 | CG7456 |
| FBgn0032296 | CG6729 |
| FBgn0032395 | CG6734 |
| FBgn0032397 | Tom70 |
| FBgn0032399 | CG6785 |
| FBgn0032475 | Sfmbt |
| FBgn0032478 | CG5458 |
| FBgn0032517 | CG7099 |
| FBgn0032586 | Tpr2 |
| FBgn0032640 | Sgt |
| FBgn0032646 | CG6412 |
| FBgn0032701 | CG10341 |
| FBgn0032704 | Jwa |
| FBgn0032707 | CG10348 |
| FBgn0032728 | Tango6 |
| FBgn0032730 | CG10431 |
| FBgn0032798 | CG10132 |
| FBgn0032876 | Cen |
| FBgn0032886 | CG9328 |
| FBgn0033050 | Pngl |
| FBgn0033052 | SCAP |
| FBgn0033055 | tbce |
| FBgn0033177 | CG11141 |
| FBgn0033199 | CG17985 |
| FBgn0033259 | CG11210 |
| FBgn0033266 | Socs44A |
| FBgn0033317 | CG8635 |
| FBgn0033337 | CG8272 |
| FBgn0033356 | CG8229 |
| FBgn0033376 | CG8777 |
| FBgn0033421 | CG1888 |
| FBgn0033438 | Mmp2 |
| FBgn0033466 | Pal1 |
| FBgn0033473 | CG12128 |
| FBgn0033504 | CAP |
| FBgn0033540 | Elp2 |
| FBgn0033569 | CG12942 |
| FBgn0033607 | CG9062 |
| FBgn0033638 | CG9005 |
| FBgn0033639 | CG9003 |
| FBgn0033652 | ths |
| FBgn0033657 | Sln |
| FBgn0033766 | CG8771 |
| FBgn0033812 | Pex13 |
| FBgn0033846 | mip120 |
| FBgn0033899 | CG13016 |
| FBgn0033911 | VGAT |
| FBgn0033916 | CG8494 |
| FBgn0034068 | casp |
| FBgn0034113 | CG8060 |
| FBgn0034186 | CG8950 |
| FBgn0034223 | Tes |
| FBgn0034230 | CG4853 |
| FBgn0034237 | eIF3-S9 |
| FBgn0034351 | CG5190 |
| FBgn0034420 | CG10737 |
| FBgn0034432 | CG7461 |
| FBgn0034442 | CG11257 |
| FBgn0034476 | Toll-7 |
| FBgn0034498 | CG16868 |
| FBgn0034504 | CG8929 |
| FBgn0034529 | CG16742 |
| FBgn0034540 | Lrt |
| FBgn0034572 | CG9346 |
| FBgn0034641 | mahj |
| FBgn0034704 | CG6758 |
| FBgn0034707 | MED16 |
| FBgn0034734 | CG4554 |
| FBgn0034792 | CG3499 |
| FBgn0034853 | Ice1 |
| FBgn0034958 | CG3907 |
| FBgn0034971 | CG3209 |
| FBgn0034989 | CG3356 |
| FBgn0035019 | Ir60e |
| FBgn0035023 | itp |
| FBgn0035047 | Pof |
| FBgn0035101 | p130CAS |
| FBgn0035111 | CG16940 |
| FBgn0035137 | CG1233 |
| FBgn0035165 | CG13887 |
| FBgn0035228 | CG12091 |
| FBgn0035232 | CG12099 |
| FBgn0035237 | CG13917 |
| FBgn0035246 | CG13928 |
| FBgn0035253 | CG7971 |
| FBgn0035264 | Oseg4 |
| FBgn0035285 | CG12025 |
| FBgn0035308 | CG15822 |
| FBgn0035333 | CG1317 |
| FBgn0035347 | CG33232 |
| FBgn0035357 | MEP-1 |
| FBgn0035383 | CG2107 |
| FBgn0035416 | gry |
| FBgn0035437 | CG11526 |
| FBgn0035455 | CG10862 |
| FBgn0035473 | mge |
| FBgn0035498 | Fit1 |
| FBgn0035519 | CG1309 |
| FBgn0035532 | CG15014 |
| FBgn0035558 | CG11357 |
| FBgn0035574 | RhoGEF64C |
| FBgn0035586 | CG10671 |
| FBgn0035688 | CG10289 |
| FBgn0035689 | CG7376 |
| FBgn0035704 | CG10144 |
| FBgn0035713 | velo |
| FBgn0035763 | CG8602 |
| FBgn0035771 | sec63 |
| FBgn0035850 | Atg18 |
| FBgn0035945 | CG5026 |
| FBgn0035953 | CG5087 |
| FBgn0035986 | CG4022 |
| FBgn0035987 | CG3689 |
| FBgn0036038 | defl |
| FBgn0036180 | CG6091 |
| FBgn0036271 | Pbgs |
| FBgn0036299 | Tsf2 |
| FBgn0036309 | Hip1 |
| FBgn0036386 | CG8833 |
| FBgn0036389 | ssp2 |
| FBgn0036402 | CG6650 |
| FBgn0036494 | Toll-6 |
| FBgn0036510 | CG7427 |
| FBgn0036518 | RhoGAP71E |
| FBgn0036522 | CG7372 |
| FBgn0036574 | CG16838 |
| FBgn0036584 | CG13054 |
| FBgn0036666 | TSG101 |
| FBgn0036684 | CG3764 |
| FBgn0036685 | CG6664 |
| FBgn0036714 | CG7692 |
| FBgn0036715 | Cad74A |
| FBgn0036725 | CG18265 |
| FBgn0036734 | CG7564 |
| FBgn0036762 | CG7430 |
| FBgn0036770 | Prestin |
| FBgn0036843 | CG6812 |
| FBgn0036888 | CG9330 |
| FBgn0036915 | Prp3 |
| FBgn0036934 | sNPF-R |
| FBgn0036958 | CG17233 |
| FBgn0036974 | eRF1 |
| FBgn0037073 | CG7338 |
| FBgn0037081 | barc |
| FBgn0037084 | Syx6 |
| FBgn0037093 | Cdk12 |
| FBgn0037094 | CG7611 |
| FBgn0037108 | CG11306 |
| FBgn0037135 | CG7414 |
| FBgn0037213 | CG12581 |
| FBgn0037240 | Cont |
| FBgn0037252 | CG14650 |
| FBgn0037270 | CG9769 |
| FBgn0037299 | CG1115 |
| FBgn0037332 | Hcs |
| FBgn0037363 | CG1347 |
| FBgn0037382 | Hpr1 |
| FBgn0037442 | CG10277 |
| FBgn0037466 | CG1965 |
| FBgn0037536 | CG2698 |
| FBgn0037549 | CG7878 |
| FBgn0037624 | CG8223 |
| FBgn0037655 | CG11984 |
| FBgn0037660 | beag |
| FBgn0037689 | CG8135 |
| FBgn0037780 | CG3925 |
| FBgn0037814 | CG6325 |
| FBgn0037847 | SelR |
| FBgn0037890 | CG17734 |
| FBgn0037915 | CG6790 |
| FBgn0038016 | MBD-R2 |
| FBgn0038039 | CG5196 |
| FBgn0038220 | CG12207 |
| FBgn0038286 | CG6966 |
| FBgn0038293 | CG6904 |
| FBgn0038300 | CG4203 |
| FBgn0038344 | CG5205 |
| FBgn0038385 | CG4221 |
| FBgn0038400 | CG5903 |
| FBgn0038418 | pad |
| FBgn0038488 | m-cup |
| FBgn0038532 | CG14322 |
| FBgn0038597 | CG8064 |
| FBgn0038638 | CG7702 |
| FBgn0038651 | CG14299 |
| FBgn0038659 | endoA |
| FBgn0038744 | CG4733 |
| FBgn0038745 | CG4538 |
| FBgn0038855 | CG5745 |
| FBgn0038880 | SIFR |
| FBgn0038889 | CG7922 |
| FBgn0038947 | Sar1 |
| FBgn0038968 | CG12499 |
| FBgn0039016 | Dcr-1 |
| FBgn0039117 | tst |
| FBgn0039120 | Nup98-96 |
| FBgn0039183 | Dis3 |
| FBgn0039212 | Syx18 |
| FBgn0039213 | atl |
| FBgn0039223 | CG5805 |
| FBgn0039227 | polybromo |
| FBgn0039260 | Smg6 |
| FBgn0039261 | CG6422 |
| FBgn0039286 | dan |
| FBgn0039303 | CG11857 |
| FBgn0039430 | CG5455 |
| FBgn0039451 | CG6420 |
| FBgn0039507 | mrt |
| FBgn0039508 | CG3368 |
| FBgn0039555 | mRpS22 |
| FBgn0039588 | CG12413 |
| FBgn0039600 | CG1646 |
| FBgn0039631 | Sirt7 |
| FBgn0039714 | CG7816 |
| FBgn0039727 | CG15523 |
| FBgn0039737 | CG7920 |
| FBgn0039767 | CG2218 |
| FBgn0039773 | CG2224 |
| FBgn0039804 | CG15544 |
| FBgn0039863 | CG1815 |
| FBgn0039929 | CG11076 |
| FBgn0039944 | CG17162 |
| FBgn0040079 | pkaap |
| FBgn0040268 | Top3alpha |
| FBgn0040271 | Sulf1 |
| FBgn0040273 | Spt5 |
| FBgn0040281 | Aplip1 |
| FBgn0040283 | SMC1 |
| FBgn0040305 | MTF-1 |
| FBgn0040319 | Gclc |
| FBgn0040372 | G9a |
| FBgn0041161 | blue |
| FBgn0042138 | CG18815 |
| FBgn0043070 | MESK2 |
| FBgn0044452 | Atg2 |
| FBgn0045842 | yuri |
| FBgn0050087 | CG30087 |
| FBgn0050115 | GEFmeso |
| FBgn0050122 | CG30122 |
| FBgn0050183 | CG30183 |
| FBgn0050479 | CG30479 |
| FBgn0050496 | CG30496 |
| FBgn0051038 | CG31038 |
| FBgn0051064 | CG31064 |
| FBgn0051156 | CG31156 |
| FBgn0051301 | CG31301 |
| FBgn0051357 | CG31357 |
| FBgn0051363 | Jupiter |
| FBgn0051467 | CG31467 |
| FBgn0051481 | pb |
| FBgn0051510 | CG31510 |
| FBgn0051612 | CG31612 |
| FBgn0051635 | CG31635 |
| FBgn0051694 | CG31694 |
| FBgn0051712 | CG31712 |
| FBgn0051793 | CG31793 |
| FBgn0051897 | CG31897 |
| FBgn0051935 | CG31935 |
| FBgn0052113 | CG32113 |
| FBgn0052264 | CG32264 |
| FBgn0052380 | SMSr |
| FBgn0052425 | CG32425 |
| FBgn0052473 | CG32473 |
| FBgn0052532 | CG32532 |
| FBgn0052591 | CG32591 |
| FBgn0052594 | be |
| FBgn0052626 | CG32626 |
| FBgn0052672 | Atg8a |
| FBgn0052675 | Tango5 |
| FBgn0052702 | CG32702 |
| FBgn0053095 | CG33095 |
| FBgn0053111 | CG33111 |
| FBgn0053113 | Rtnl1 |
| FBgn0053144 | CG33144 |
| FBgn0053207 | pxb |
| FBgn0061469 | Ube3a |
| FBgn0063649 | CG6006 |
| FBgn0067102 | GlcT-1 |
| FBgn0067779 | dbr |
| FBgn0083942 | CG34106 |
| FBgn0083969 | CG34133 |
| FBgn0085397 | Fili |
| FBgn0085407 | Pvf3 |
| FBgn0085408 | Shroom |
| FBgn0085410 | TrissinR |
| FBgn0086347 | Myo31DF |
| FBgn0086694 | Bre1 |
| FBgn0086736 | GckIII |
| FBgn0086901 | cv-c |
| FBgn0243511 | psidin |
| FBgn0250791 | Snap |
| FBgn0250906 | Pgk |
| FBgn0259111 | Ndae1 |
| FBgn0259152 | Clbn |
| FBgn0259166 | CG42271 |
| FBgn0259168 | mnb |
| FBgn0259170 | alpha-Man-I |
| FBgn0259172 | rn |
| FBgn0259173 | corn |
| FBgn0259175 | ome |
| FBgn0259176 | bun |
| FBgn0259199 | CG42303 |
| FBgn0259213 | CG42313 |
| FBgn0259221 | CG42321 |
| FBgn0259222 | CG42322 |
| FBgn0259227 | CG42327 |
| FBgn0259243 | Pka-R1 |
| FBgn0259785 | pzg |
| FBgn0259923 | 4-Sep |
| FBgn0259984 | kuz |
| FBgn0260439 | Pp2A-29B |
| FBgn0260462 | CG12163 |
| FBgn0260789 | mxc |
| FBgn0260933 | rempA |
| FBgn0260935 | ird1 |
| FBgn0260936 | scny |
| FBgn0260972 | alc |
| FBgn0261053 | Cad86C |
| FBgn0261108 | Atg13 |
| FBgn0261243 | Psa |
| FBgn0261387 | CG17528 |
| FBgn0261461 | RhoGAP18B |
| FBgn0261477 | slim |
| FBgn0261509 | haf |
| FBgn0261554 | CG42672 |
| FBgn0261555 | CG42673 |
| FBgn0261556 | CG42674 |
| FBgn0261564 | CG42678 |
| FBgn0261565 | Lmpt |
| FBgn0261570 | CG42684 |
| FBgn0261573 | CoRest |
| FBgn0261641 | CG42724 |
| FBgn0261648 | salm |
| FBgn0261703 | gce |
| FBgn0261938 | mtRNApol |
| FBgn0261953 | TfAP-2 |
| FBgn0261988 | Gprk2 |
| FBgn0262018 | CadN2 |
| FBgn0262057 | Spn77Ba |
| FBgn0262125 | Sec23 |
| FBgn0262519 | Mi-2 |
| FBgn0262527 | wah |
| FBgn0262559 | Mdh2 |
| FBgn0262579 | Ect4 |
| FBgn0262617 | CG43143 |
| FBgn0262707 | CTPsyn |
| FBgn0262714 | Sap130 |
| FBgn0262719 | CG43163 |
| FBgn0262869 | Gfrl |
| FBgn0262975 | cnc |
| FBgn0263144 | bin3 |
| FBgn0263391 | hts |
| FBgn0263603 | Zn72D |
| FBgn0263705 | Myo10A |
| FBgn0263772 | CG43689 |
| FBgn0263846 | CG43707 |
| FBgn0263968 | nonC |
| FBgn0263998 | Ack-like |
| FBgn0264326 | DNApol-epsilon |
| FBgn0264357 | SNF4Agamma |
| FBgn0264542 | CG43921 |
| FBgn0264753 | Rgk1 |
| FBgn0264835 | CR44043 |
| FBgn0264908 | pHCl |
| FBgn0264962 | Inr-a |
| FBgn0265002 | CG44153 |
| FBgn0265003 | koi |
| FBgn0265416 | Neto |
| FBgn0265434 | zip |
| FBgn0265464 | Traf6 |
| FBgn0265512 | mlt |
| FBgn0265726 | NnaD |

**Supplemental Table S7. Number of alleles per gene of the sex determination hierarchy in a random 50% subset of CEGS lines.**

| **gene** | **FBname** | **CGnum** | **CEGS (assume ref)** | **CEGS (imputed)** |
| --- | --- | --- | --- | --- |
| her | FBgn0001185 | CG4694 | 17 | 11 |
| Spf45 | FBgn0086683 | CG17540 | 8 | 7 |
| ix | FBgn0001276 | CG13201 | 6 | 6 |
| fl(2)d | FBgn0000662 | CG6315 | 14 | 8 |
| tra2 | FBgn0003742 | CG10128 | 11 | 10 |
| Psi | FBgn0014870 | CG8912 | 19 | 7 |
| vir | FBgn0003977 | CG3496 | 30 | 23 |
| tra | FBgn0003741 | CG16724 | 7 | 6 |
| mub | FBgn0262737 | CG7437 | 11 | 6 |
| dsx | FBgn0000504 | CG11094 | 13 | 3 |
| ps | FBgn0261552 | CG42670 | 18 | 9 |
| Rbp1 | FBgn0260944 | CG17136 | 4 | NA |
| sqd | FBgn0263396 | CG16901 | 9 | 6 |
| B52 | FBgn0004587 | CG10851 | 11 | 3 |
| fru | FBgn0004652 | CG14307 | 34 | 22 |
| snf | FBgn0003449 | CG4528 | 12 | 11 |
| Sxl | FBgn0264270 | CG43770 | 6 | 5 |
| Yp2 | FBgn0005391 | CG2979 | 13 | 9 |
| Yp1 | FBgn0004045 | CG2985 | 14 | 6 |
| Yp3 | FBgn0004047 | CG11129 | 20 | 11 |

NA: Indicates that depth of coverage was insufficient to estimate the number of alleles.

**Supplemental Table S8. Genes added to the sex hierarchy GRN that showed enrichment for chromatin binding and helicase activity.**

| **GO_molecular_function_cat Category** | **primary_fbgn** | **symbol** |
| --- | --- | --- |
| chromatin binding | FBgn0000629 | E(z) |
| chromatin binding | FBgn0001325 | Kr |
| chromatin binding | FBgn0002781 | mod(mdg4) |
| chromatin binding | FBgn0002914 | Myb |
| chromatin binding | FBgn0004374 | neb |
| chromatin binding | FBgn0004861 | ph-p |
| chromatin binding | FBgn0005616 | msl-2 |
| chromatin binding | FBgn0011741 | Arp6 |
| chromatin binding | FBgn0013263 | Trl |
| chromatin binding | FBgn0014340 | mof |
| chromatin binding | FBgn0024807 | DIP1 |
| chromatin binding | FBgn0025802 | Sbf |
| chromatin binding | FBgn0030082 | HP1b |
| chromatin binding | FBgn0032475 | Sfmbt |
| chromatin binding | FBgn0034853 | Ice1 |
| chromatin binding | FBgn0035047 | Pof |
| chromatin binding | FBgn0035357 | MEP-1 |
| chromatin binding | FBgn0039227 | polybromo |
| chromatin binding | FBgn0040283 | SMC1 |
| chromatin binding | FBgn0259785 | pzg |
| chromatin binding | FBgn0261573 | CoRest |
| chromatin binding | FBgn0262519 | Mi-2 |
| helicase activity | FBgn0000212 | brm |
| helicase activity | FBgn0001330 | kz |
| helicase activity | FBgn0002906 | Blm |
| helicase activity | FBgn0015075 | Ddx1 |
| helicase activity | FBgn0027375 | RecQ5 |
| helicase activity | FBgn0030354 | Upf1 |
| helicase activity | FBgn0030833 | CG8915 |
| helicase activity | FBgn0035689 | CG7376 |
| helicase activity | FBgn0037549 | CG7878 |
| helicase activity | FBgn0038344 | CG5205 |
| helicase activity | FBgn0038889 | CG7922 |
| helicase activity | FBgn0039016 | Dcr-1 |
| helicase activity | FBgn0039117 | tst |
| helicase activity | FBgn0262519 | Mi-2 |

**Supplemental Table S9. DSPR isoform level factor analysis.**

| Factor ID | Isoform |
| --- | --- |
| 0 | Rm62, vir, ps, her |
| 1 | Sxl_PA, Sxl_PAB, SXL_PC, Sxl_PE, Sxl_PG, Sxl_PH, Sxl_PJ, Sxl_PN, Sxl_PO, Sxl_PP, Sxl_PR, Sxl_PT, Sxl_PW, Sxl_PX, Sxl_PY |
| 2 | fru_PA, fru_PB, fru_PC, fru_PD, fru_PE, fru_PF, fru_PG, fru_PH, fru_PI, fru_PJ, fru_PK, fru_PL, fru_PM, fru_PN, fru_PO |
| 3 | tra2_PA, tra2_PB, tra2_PC, tra2_PD, tra2_PE, tra2_PF, tra2_PG |
| 4 | B52_PA, B52_PB, B52_PC, B52_PM, B52_PN, B52_PO |
| 5 | mub_PA, mub_PC, mub_PG, mub_PH, mub_PL, mub_PM |
| 6 | sq_PA, sqd_PB, sqd_PC, sqd_PD, sqd_PE |
| 7 | Sxl_PAA, Sxl_PB, Sxl_PF, Sxl_PM |
| 8 | mub_PD, mub_PE, mub_PF, mub_PI, mub_PK |
| 9 | B52_PD, B52_PF, B52_PI, B52_PK |
| 10 | fl(2)d_PA, fl(2)d_PB, fl(2)d_PC, fl(2)d_PD |
| 11 | Sxl_PK, Sxl_PQ, Sxl_PZ |
| 12 | Spf45_PA, Spf45_PB, Spf45_PC |
| 13 | Rbp1_PA, Rbp1_PD |
| 14 | Yp3, Yp2, Yp1 |
| 15 | tra_PA, ix |
| 16 | snf, Psi |

**Supplemental Table S10. DSPR gene level factor analysis.**

| **Factor ID** | **Gene** |
| --- | --- |
| 0 | Spf45 |
| 1 | Psi, Rdp1, fru, ps |
| 2 | Yp1, Yp2, Yp3 |
| 3 | ix, tra |
| 4 | her, mub, sqd |
| 5 | fl(2)d, snf |
| 6 | B52, vir |
| 7 | Rm62 |
| 8 | Sxl, tra2 |

**Supplemental Table S11. CEGS exonic regions factor analysis.**

| **Factor ID** | **Exonic Region** |
| --- | --- |
| 0 | Sxl_F14586_SI, snf_S13633_SI, fl_2_d_S40438_SI, vir_S46612_SI, dsx_S50110_SI, ps_S50891_SI, fru_S55899_SI |
| 1 | Sxl_F14589_SI, mub_F33103_SI, mub_F33107_SI, mub_F33109_SI, fl_2_d_F40441_SI, fl_2_d_F40442_SI, Psi_F42483_SI, Psi_F42487_SI, sqd_F53304_SI, B52_F53322_SI, Sxl_S14581_SI, Sxl_S14588_SI, mub_S33093_SI, mub_S33096_SI, mub_S33100_SI, mub_S33101_SI, mub_S33102_SI, mub_S33104_SI, Psi_S42484_SI, Psi_S42485_SI, Psi_S42486_SI, vir_S46613_SI, B52_S53321_SI, fru_S55894_SI, fru_S55895_SI, fru_S55896_SI, fru_S55897_SI, her_S8954_SI |
| 2 | Sxl_F14580_SI, Sxl_F14582_SI, Sxl_F14587_SI, vir_F46610_SI, ps_F50887_SI, ps_F50889_SI, ps_F50897_SI, sqd_F53296_SI, sqd_F53299_SI, Sxl_S14583_SI, ps_S50892_SI, ps_S50893_SI, ps_S50895_SI, ps_S50896_SI, sqd_S53300_SI, sqd_S53301_SI, sqd_S53302_SI |
| 3 | Yp2_F15820_SI, Yp2_F15821_SI, Yp1_S15822_SI, Yp1_S15823_SI, Yp3_S17610_SI, Yp3_S17611_SI, Yp3_S17612_SI |
| 4 | Spf45_F11583_SI, tra_F30270_SI, tra2_F40928_SI, Spf45_S11582_SI, ix_S38844_SI |
| 5 | Psi_F42482_SI, dsx_F50104_SI, dsx_F50106_SI, Rbp1_F51767_SI, B52_F53325_SI |
| 6 | snf_S13634_SI, sqd_S53297_SI, sqd_S53298_SI, B52_S53324_SI |
| 7 | fru_F55889_SI, fru_F55892_SI, fru_F55906_SI, fru_S55890_SI, fru_S55891_SI |
| 8 | mub_S33106_SI, fl_2_d_S40439_SI, B52_S53320_SI, her_S8955_SI |
| 9 | Sxl_F14585_SI, tra2_F40929_SI, B52_F53319_SI, Rbp1_S51766_SI |
| 10 | tra2_F40932_SI, tra2_F40933_SI |
| 11 | sqd_S53303_SI, fru_S55907_SI |
| 12 | fl_2_d_S40440_SI, vir_S46611_SI |
| 13 | Spf45_S11584_SI, mub_S33105_SI |
| 14 | mub_S33108_SI |
| 15 | tra_F30271_SI, dsx_S50108_SI, B52_S53323_SI |
| 16 | vir_S46614_SI |
| 17 | her_F8956_SI |

**Supplemental Table S12. CEGS gene level factor analysis.**

| **Factor ID** | **Gene** |
| --- | --- |
| 0 | snf |
| 1 | Psi, Sxl, fl(2)d, fru, mub |
| 2 | Yp1, Yp2, Yp3 |
| 3 | ps, sqd |
| 4 | tra |
| 5 | tra2 |
| 6 | dsx |
| 7 | Rbp1 |
| 8 | her |
| 9 | Spf45 |
| 10 | vir |
| 11 | B52 |
| 12 | ix |

**Supplemental Table S13. DSPR isoform level modulated modularity clustering (MMC).**

| **Module ID** | **Isoform** |
| --- | --- |
| 1 | mub_PE, mub_PF |
| 2 | B52_PM, B52_PO |
| 3 | fl_2_d_PB, fl_2_d_PC, fl_2_d_PD |
| 4 | fru_PB, fru_PF |
| 5 | mub_PD, mub_PK |
| 6 | Sxl_PK, Sxl_PQ, Sxl_PZ |
| 7 | Sxl_PAA, Sxl_PB, Sxl_PF, Sxl_PM |
| 8 | mub_PG, mub_PM |
| 9 | sqd_PC, sqd_PE, sqd_PB |
| 10 | B52_PD, B52_PI, B52_PF, B52_PK |
| 11 | mub_PA, mub_PC, mub_PH, mub_PL |
| 12 | Spf45_PB, Spf45_PC |
| 13 | tra2_PA, tra2_PF, tra2_PG, tra2_PC, tra2_PD, tra2_PE |
| 14 | B52_PA, B52_PC, B52_PN, B52_PB |
| 15 | Sxl_PA, Sxl_PE, Sxl_PG, Sxl_PJ, Sxl_PC, Sxl_PN, Sxl_PR, Sxl_PP, Sxl_PW |
| 16 | Sxl_PD, Sxl_PL, Sxl_PT, Sxl_PAB, Sxl_PH, Sxl_PO, Sxl_PY |
| 17 | fru_PA, fru_PG, fru_PH, fru_PK, fru_PO, ps, tra2_PB |
| 18 | fru_PC, fru_PE, fru_PI, fru_PJ, fru_PL, fru_PM, fru_PN, fru_PD, Psi, vir, snf |
| 19 | Rbp1_PD, Rbp1_PA, Spf45_PA, sqd_PD |
| 20 | Yp3, Yp2, Yp1, her, tra_PA, fl_2_d_PA, ix, Rm62 |
| 21 | Sxl_PX |
| 22 | mub_PI |
| 23 | sqd_PA |

**Supplemental Table S14. CEGS exon level modulated modularity clustering (MMC).**

| **Module** | **Exonic Region** |
| --- | --- |
| 1 | Yp3_S17611_SI, Yp2_F15820_SI, Yp3_S17610_SI, Yp2_F15821_SI, Yp3_S17612_SI, Yp1_S15822_SI, Yp1_S15823_SI |
| 2 | fru_S55895_SI, Sxl_F14589_SI, fru_S55890_SI, fru_F55906_SI, Sxl_F14580_SI, fru_S55894_SI, Sxl_F14585_SI, fru_S55896_SI, fru_S55897_SI, Sxl_S14581_SI, snf_S13633_SI, fl_2_d_F40442_SI, Sxl_F14587_SI, Sxl_S14588_SI, Sxl_F14582_SI, vir_S46613_SI, Sxl_S14583_SI, fl_2_d_F40441_SI, fl_2_d_S40439_SI, vir_S46612_SI, ix_S38844_SI, fru_F55889_SI, fru_S55907_SI, fru_F55892_SI, her_S8954_SI, tra2_F40928_SI, vir_F46610_SI, fl_2_d_S40440_SI, Spf45_F11583_SI, snf_S13634_SI, fru_S55891_SI, Sxl_F14586_SI, fl_2_d_S40438_SI, her_F8956_SI, vir_S46611_SI, Spf45_S11582_SI, tra2_F40929_SI, tra_F30270_SI, tra2_F40933_SI, Spf45_S11584_SI, tra_F30271_SI, vir_S46614_SI, tra2_F40932_SI, fru_S55899_SI, her_S8955_SI |

# Figures


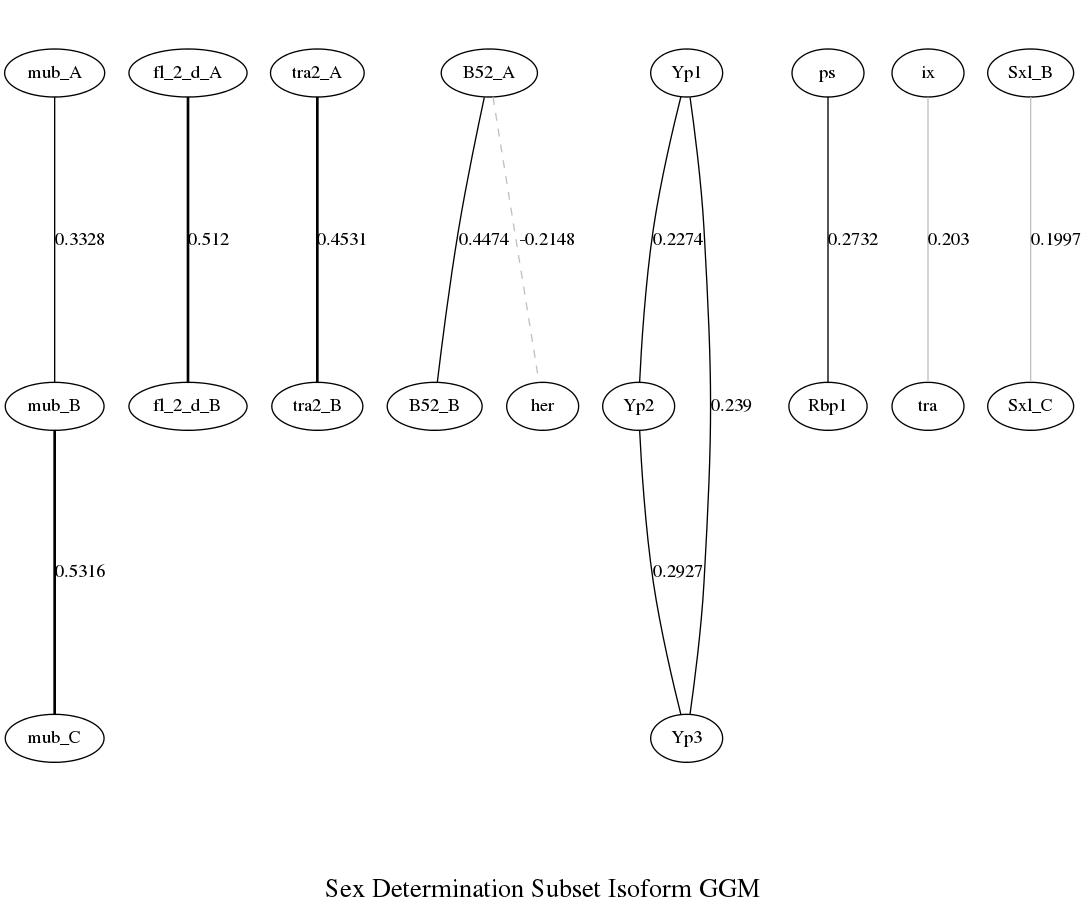


**Supplementary Figure S1. Graphical Gaussian network of genes in the sex determination hierarchy (DSPR Collapsed Isoforms).** A GGN was constructed using only the combined isoforms in the sex hierarchy. Isoforms are represented as nodes and relationships between genes are edges. Numbers are the estimated partial correlation coefficient between genes. Solid (dashed) lines represent positive (negative) partial correlations. Only edges passing an FDR threshold (FDR 0.2) are shown.


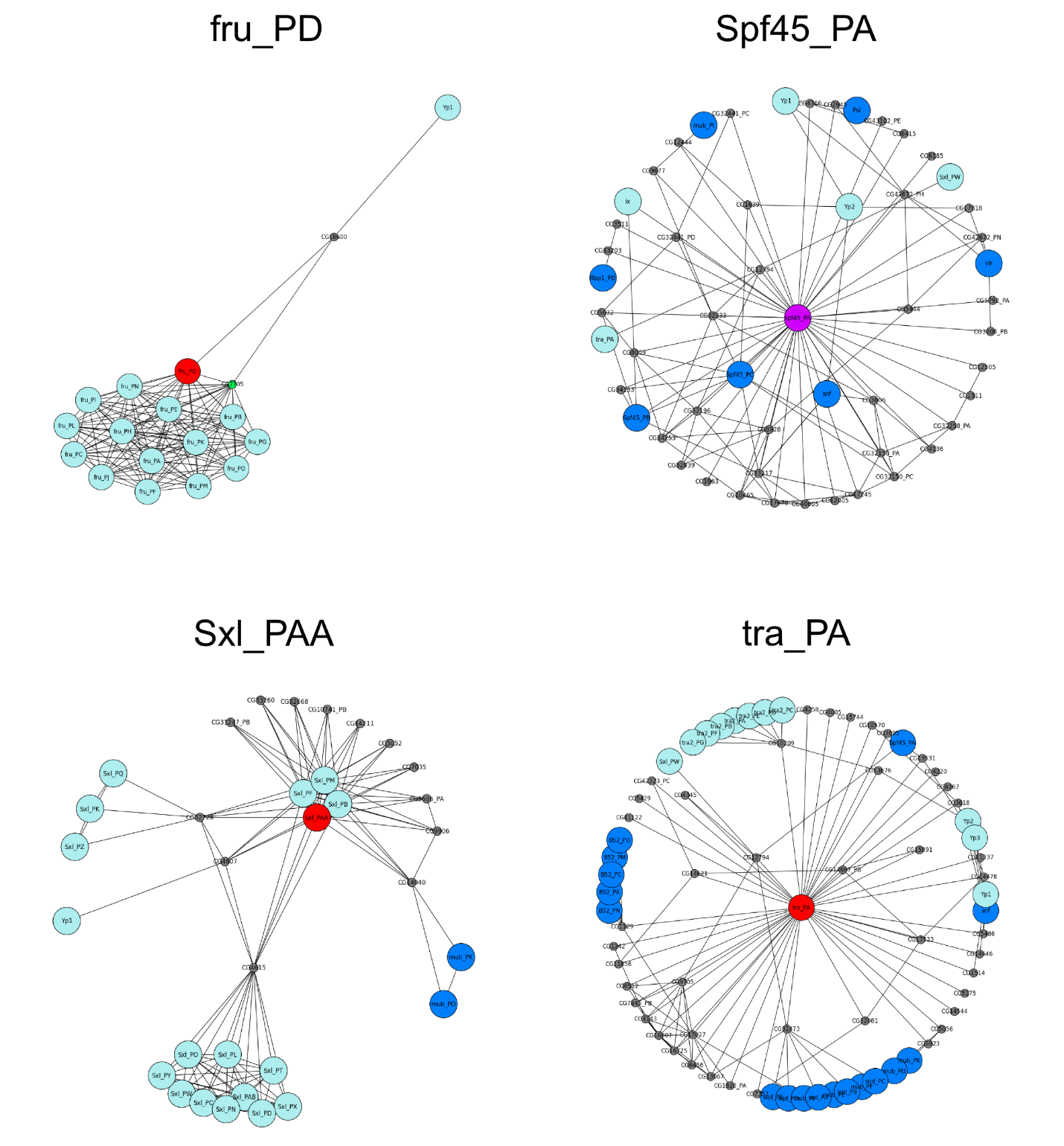


**Supplementary Figure S2. Examples of secondary neighborhood structure from a genome-wide graphical Gaussian network.** Secondary network structures for individual genes in the sex hierarchy were visualized. Genes are colored as follows; red/purple (current focal gene), grey (not associated with the sex hierarchy), light blue (in the sex hierarchy), dark blue (splicing factors associated with the sex hierarchy). Grey genes 2-steps out from the focal gene were not visualized to reduce image complexity. (A-B) Gene isoforms tend to cluster together in the same neighborhood. (C-D) Other genes are more connected to other members of the sex hierarchy


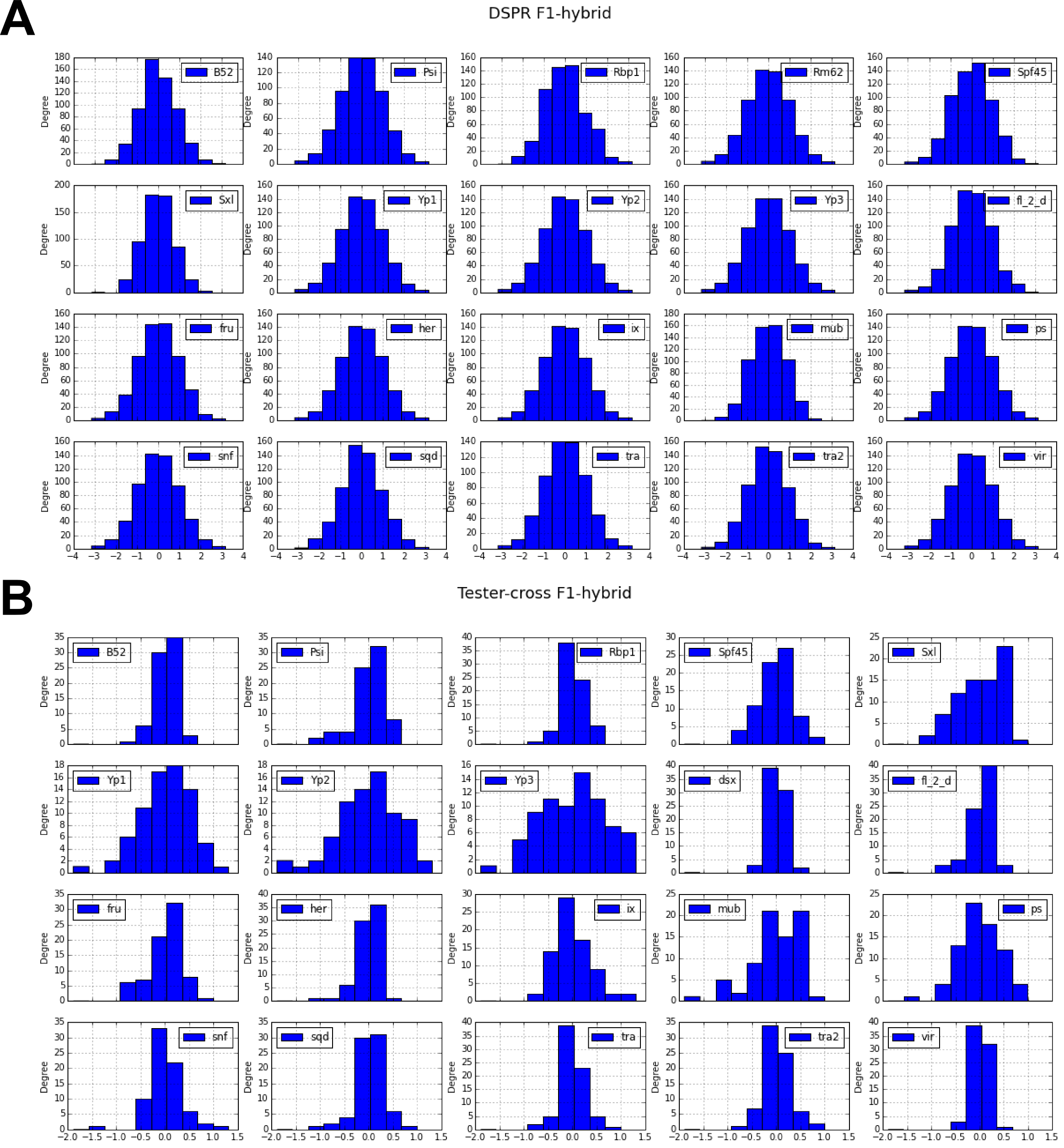


**Supplementary Figure S3. Distribution of genes in the sex hierarchy.** Plotted are the distribution of genes in the sex hierarchy, DSPR (A) or CEGS population (B), after normalization and transformation.


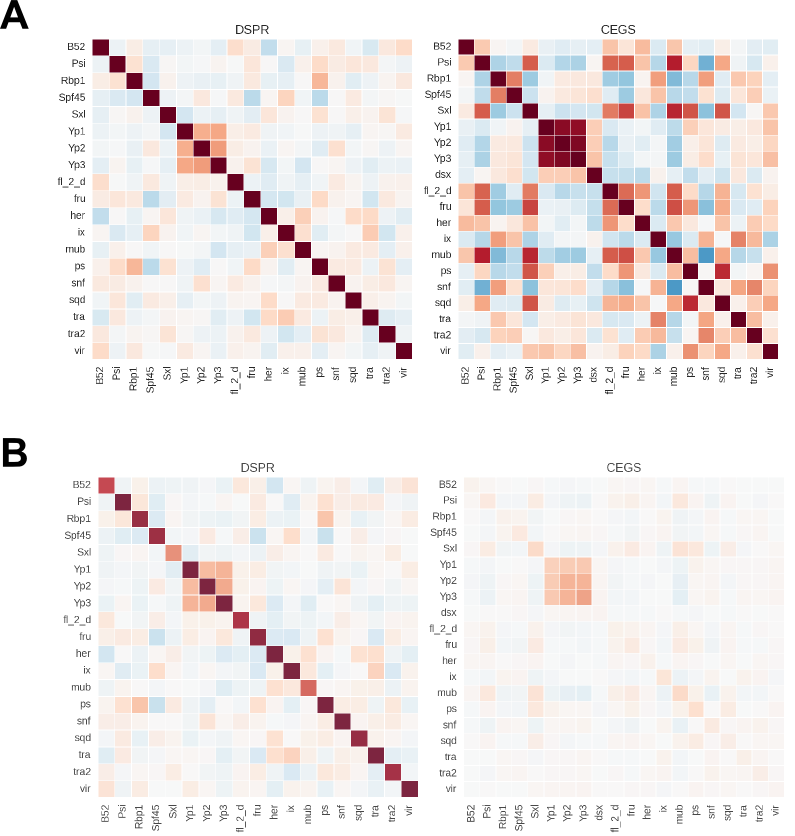


**Supplementary Figure S4. Correlation and covariance matrices for genes in the sex hierarchy.** Heat maps show the correlation (A) and covariance (B) structure among genes in the sex hierarchy for the DSPR and CEGS populations. Correlation structure is stronger in the CEGS population (A) while variance shown as covariance structure (B) is stronger in the DSPR. Colors for all plots are scaled the same, with dark red being values close to 1 and dark blue as values close to -1.
